# Receptor-Targeted Nipah Virus Glycoproteins Improve Cell-Type Selective Gene Delivery and Reveal a Preference for Membrane-Proximal Cell Attachment

**DOI:** 10.1371/journal.ppat.1005641

**Published:** 2016-06-09

**Authors:** Ruben R. Bender, Anke Muth, Irene C. Schneider, Thorsten Friedel, Jessica Hartmann, Andreas Plückthun, Andrea Maisner, Christian J. Buchholz

**Affiliations:** 1 Molecular Biotechnology and Gene Therapy, Paul-Ehrlich-Institut, Langen, Germany; 2 Department of Biochemistry, University of Zurich, Zurich, Switzerland; 3 Institute for Virology (BMFZ), Philipps-University Marburg, Marburg, Germany; Georgia State University, UNITED STATES

## Abstract

Receptor-targeted lentiviral vectors (LVs) can be an effective tool for selective transfer of genes into distinct cell types of choice. Moreover, they can be used to determine the molecular properties that cell surface proteins must fulfill to act as receptors for viral glycoproteins. Here we show that LVs pseudotyped with receptor-targeted Nipah virus (NiV) glycoproteins effectively enter into cells when they use cell surface proteins as receptors that bring them closely enough to the cell membrane (less than 100 Å distance). Then, they were flexible in receptor usage as demonstrated by successful targeting of EpCAM, CD20, and CD8, and as selective as LVs pseudotyped with receptor-targeted measles virus (MV) glycoproteins, the current standard for cell-type specific gene delivery. Remarkably, NiV-LVs could be produced at up to two orders of magnitude higher titers compared to their MV-based counterparts and were at least 10,000-fold less effectively neutralized than MV glycoprotein pseudotyped LVs by pooled human intravenous immunoglobulin. An important finding for NiV-LVs targeted to Her2/*neu* was an about 100-fold higher gene transfer activity when particles were targeted to membrane-proximal regions as compared to particles binding to a more membrane-distal epitope. Likewise, the low gene transfer activity mediated by NiV-LV particles bound to the membrane distal domains of CD117 or the glutamate receptor subunit 4 (GluA4) was substantially enhanced by reducing receptor size to below 100 Å. Overall, the data suggest that the NiV glycoproteins are optimally suited for cell-type specific gene delivery with LVs and, in addition, for the first time define which parts of a cell surface protein should be targeted to achieve optimal gene transfer rates with receptor-targeted LVs.

## Author Summary

Pseudotyping of lentiviral vectors (LVs) with glycoproteins from other enveloped viruses has not only often been revealing in mechanistic studies of particle assembly and entry, but is also of practical importance for gene delivery. LVs pseudotyped with engineered glycoproteins allowing free choice of receptor usage are expected to overcome current limitations in cell-type selectivity of gene transfer. Here we describe for the first time receptor-targeted Nipah virus glycoproteins as important step towards this goal. LV particles carrying the engineered Nipah virus glycoproteins were substantially more efficient in gene delivery than their state-of-the-art measles virus-based counterparts, now making the production of receptor-targeted LVs for clinical applications possible. Moreover, the data define for the first time the molecular requirements for membrane fusion with respect to the position of the receptor binding site relative to the cell membrane, a finding with implications for the molecular evolution of paramyxoviruses using proteinaceous receptors for cell entry.

## Introduction

Cell entry as first step in the viral replication cycle is initiated by the attachment of virus particles to distinct cell surface proteins. While many viral receptors have been identified, there is only limited knowledge available about the molecular requirements that cell surface proteins have to fulfill to act as entry receptors and why they have been chosen during viral evolution [1]. Paramyxoviruses encode two envelope proteins required for cell entry, the receptor attachment protein and the fusion protein (F) which mediates fusion of the viral and cellular membranes upon receptor contact. Three types of attachment proteins can be distinguished, the hemagglutinin-neuraminidase (HN), the hemagglutinin (H) and the glycoprotein (G), which in contrast to the others has no hemagglutinating function. All attachment proteins are type II membrane proteins with a membrane proximal stalk domain and a propeller-like head domain [2]. While HN proteins use sialic acid as receptor, morbillivirus H and henipavirus G recognize proteinaceous receptors. Due to this and its separated attachment and fusion functions, the measles virus (MV) H protein has been the first viral attachment protein that was successfully engineered to use a cell surface protein of choice for entry instead of its natural receptor [3].

While this approach suggested a high flexibility in receptor usage for MV, it was also of applied relevance for the engineering of tumor–specific oncolytic viruses [4] and when combined with pseudotyping for the generation of cell-type specific lentiviral vectors (LVs). With LVs as a major tool, gene therapy has developed to one of the most important technologies in modern medicine for the treatment of monogenetic diseases as well as various cancer types [5–7]. LVs mediate stable long-term expression and integration of transgenes into the genome of transduced cells. The commonly used LVs for therapeutic applications are pseudotyped with either the glycoprotein G of the vesicular stomatitis virus (VSV) or the envelope (Env) proteins of γ-retroviruses such as murine leukemia virus (MLV) or, more recently, the baboon retrovirus [8]. Since the use of all these glycoproteins result in LVs with a broad cellular tropism allowing gene transfer into a variety of cell types, further modifications in vector design have been established to restrict gene transfer into the cell type relevant for a given application.

The concept of engineering vector particle entry relies on targeting the particles to a cell surface protein of choice which is then used as entry receptor [9,10]. By picking surface proteins that are selectively expressed in a particular cell type, gene transfer can be restricted to this cell type. Natural receptor usage is destroyed through mutating specific residues in the attachment protein and the desired receptor usage is achieved through displaying a polypeptide (targeting domain) exhibiting high affinity for the targeted receptor. So far, the envelope glycoproteins from three different viruses have been successfully engineered to generate such receptor-targeted LVs. While all available receptor-targeted LVs work in principle, they also have certain disadvantages making their broad application and translation into the clinic difficult.

LVs pseudotyped with receptor-targeted Sindbis virus glycoproteins have been developed for a large variety of cell types but are limited by the non-covalent linkage of the targeting domain, the requirement for low pH membrane fusion triggering provided by efficient endocytosis of the targeted receptor as well as insufficient selectivity, which often has been compensated by the use of cell-type specific promoters [11–13]. A similar large number of cell surface proteins has been targeted by engineered MV glycoproteins, resulting in LVs exhibiting high selectivity for their target cells even when combined with the strong and ubiquitously active spleen focus forming virus (SFFV) promotor [10,14]. MV-based receptor-targeted LVs, however, can only be produced at moderate titers and are susceptible towards neutralizing antibodies induced by MV vaccination, thus preventing multiple dosing in patients. While the latter problem may be circumvented by engineered Tupaia paramyxovirus (TPMV) glycoproteins, this system turned out to be too inefficient in vector production [15].

To address the disadvantages of the MV- and TPMV-based systems, while keeping their high selectivity, we generated here for the first time receptor-targeted glycoproteins derived from a henipavirus. Nipah virus (NiV) is naturally harbored by fruit bats and can cause fatal illness in humans with respiratory and encephalitic symptoms [16]. As no vaccination programs against NiV exist, neutralizing antibodies in the human population are vanishingly small. Previous studies showed that LVs can be pseudotyped with the NiV glycoproteins resulting in vector stocks with high titers, although different cytoplasmic tail truncations were found to be optimal [17–19]. These results suggest that the NiV glycoproteins may be more suited for the generation of receptor-targeted LVs than those of MV or TPMV. Here we show, that LVs pseudotyped with the engineered NiV glycoproteins delivered genes to their target cells as selectively as MV-based receptor-targeted LVs but could be produced at substantially higher titers. Notably, receptor-targeted NiV-LVs turned out to be highly sensitive towards the position of their binding site on the targeted receptor, with membrane-proximal positions being preferred over membrane-distal ones.

## Results

### Setting up the system

**Fig 1:**
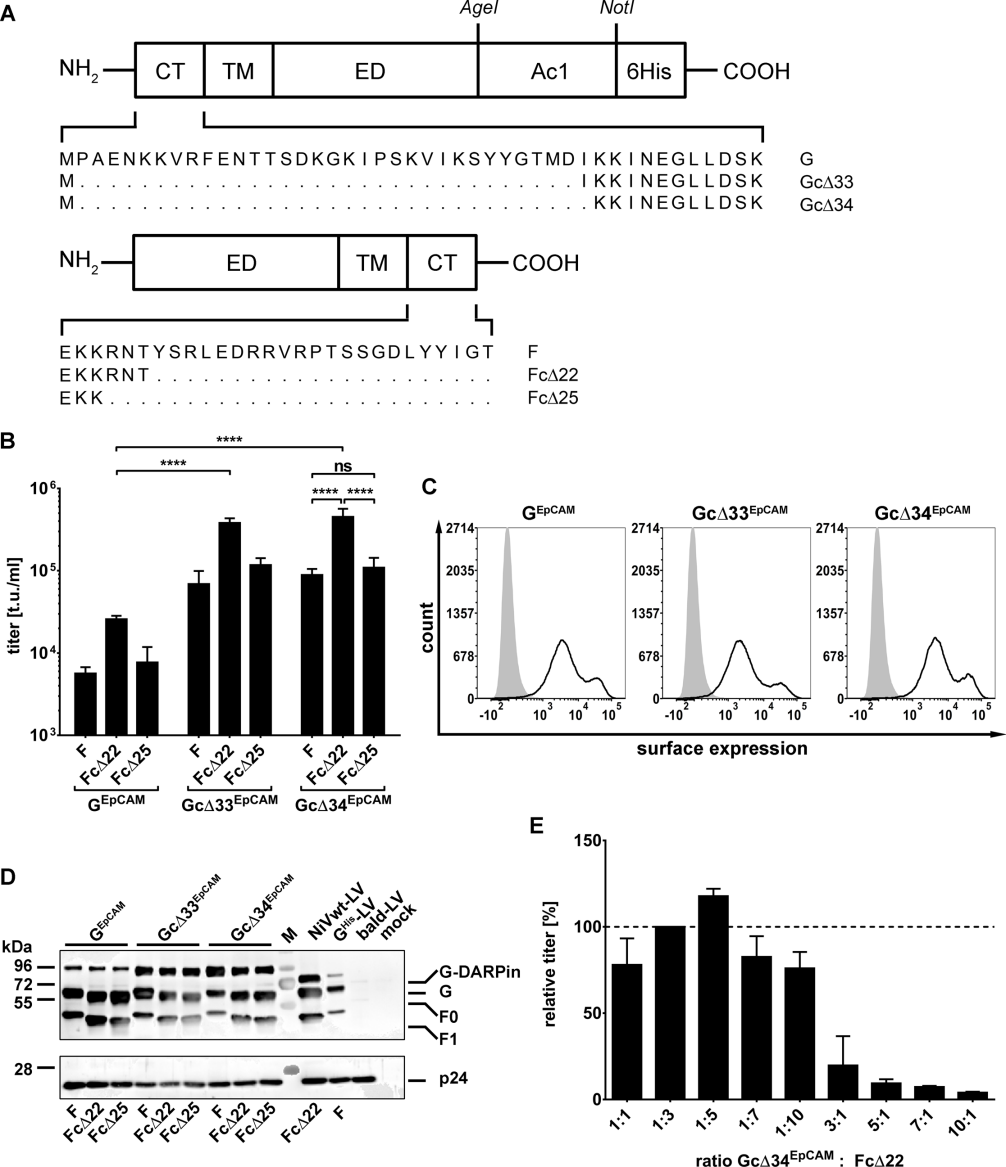
Establishing the pseudotyping of LV particles with DARPin-displaying NiV glycoproteins. (**A**) Schematic drawing of modified NiV envelope proteins. The glycoprotein G variant consisting of differently truncated cytoplasmic tails (CT), transmembrane domain (TM), ectodomain (ED), the fused EpCAM-specific DARPin Ac1 and a His tag is shown in the top row. Below, the schematic drawing of the fusion protein (F) cytoplasmic tail variants is shown. (**B**) Unconcentrated screening titers (t.u./ml) of all nine combinations of G and F protein variants titrated on CHO-EpCAM cells (n = 3; mean ± standard deviations (SD) are shown; ****, P<0.0001; ns, not significant by one-way ANOVA with Tukey's multiple comparisons test). (**C**) Surface expression of EpCAM-targeted G variants on HEK-293T cells transiently transfected with the corresponding expression plasmids (empty curves) compared to mock transfected cells (filled curves) as determined by flow cytometry. Cells were stained with PE-coupled anti-His antibody. One representative out of three experiments is shown. For quantitative data see S1 Fig. (**D**) Western blot analysis of LV particles for incorporation of G^EpCAM^, Gc∆33^EpCAM^, and Gc∆34^EpCAM^ and the three different F variants (full length F, Fc∆22 and Fc∆25). For generation of LVs used for Western blot analysis, in contrast to other vector productions, F protein with C-terminally fused AU1 tag was used in order to allow detection via an anti-AU1 antibody. Incorporation of the G variants was detected via an anti-His antibody. 2.5x10^10^ particles per sample were applied. NiV-G^His^/F (G^His^-LV) and Gc∆34^His^/Fc∆22 (NiVwt-LV) pseudotyped LVs as well as concentrated supernatant of mock transfected cells (mock) and concentrated supernatant of cells transfected with the *gag/pol* encoding plasmid pCMV∆R8.9 only (bald-LV) served as controls. M indicates the marker lane. For quantitative data see S2 Fig. (**E**) In order to optimize titers, the ratio of the amounts of the plasmids encoding the Gc∆34^EpCAM^ and the Fc∆22 protein was varied for vector production in HEK-293T cells as indicated. The produced vector stocks were titrated on CHO-EpCAM cells, and their relative titers, normalized to that obtained after transfection of the 1:3 ratio, is shown.

Previous studies showed that LVs can be pseudotyped with the NiV glycoproteins resulting in vector stocks with high titers [17–19]. While these studies showed that successful pseudotyping is achieved by cytoplasmic tail truncation of the fusion protein F, only Witting et al. (2013) [19] found that the glycoprotein G had to be truncated as well, while others came to different conclusions [17–19]. In order to generate targeted NiV-LVs, we therefore first tested the previously described cytoplasmic tail variants of NiV G and F (Fig 1A). As target receptor we chose the human epithelial cell adhesion molecule (EpCAM), a putative marker of early tumor cells [20], and fused the EpCAM-specific DARPin Ac1 [21] to the ectodomain of the G protein cytoplasmic tail variants. All nine combinations of the resulting G^EpCAM^ and F (Fig 1A) variants were assessed for their ability to mediate transfer of the *gfp* gene into CHO-EpCAM cells. CHO cells lack the natural NiV receptors ephrin-B2 and ephrin-B3 and are not susceptible for NiV. Gene transfer into CHO-EpCAM cells must therefore have been mediated by entry via human EpCAM. All NiV-LVs tested showed transduction of CHO-EpCAM cells (Fig 1B). The highest titers were obtained when the cytoplasmic tail of the F protein was truncated by 22 residues and that of the G^EpCAM^ protein by 33 or 34 amino acids, with a slight but not significant advantage for Gc∆34, resulting in unconcentrated titers of 4-5x10^5^ t.u./ml. The differences in titers were not due to differences in the cell surface expression levels of the G^EpCAM^ variants, since HEK-293T cells transfected with plasmids encoding the three different constructs showed similar high surface expression levels (Fig 1C; S1 Fig).

Next, incorporation of the G^EpCAM^ variants into LV particles was investigated. Particles pseudotyped with all nine combinations of G^EpCAM^, Gc∆33^EpCAM^ or Gc∆34^EpCAM^ with F, Fc∆22 or Fc∆25 were produced, normalized by particle numbers and analyzed via Western blot analysis. As controls, we also produced LV stocks pseudotyped with untruncated but His-tagged G/F (G^His^-LV) or with Gc∆34^His^/Fc∆22 (NiVwt-LV). All G and F variants were incorporated into vector particles (Fig 1D). When correlated to the intensities of the p24 signals, Gc∆33^EpCAM^ and Gc∆34^EpCAM^ showed substantially higher incorporation levels than G^EpCAM^ and G^His^ carrying full-length C-tails (S2 Fig), which corresponds well to the higher vector titers observed for these constructs. The combination Gc∆34^EpCAM^ and Fc∆22 was used for further vector productions.

As the ratio of G to F in the viral envelope can influence virus-cell fusion and thus LV entry, we aimed to determine the optimal ratio of plasmids. Ratios of plasmids ranging from a tenfold excess of F- over G-encoding plasmid to a tenfold excess of G over F were tested. Overall, higher amounts of the Gc∆34^EpCAM^ encoding plasmid reduced LV titers (Fig 1E). The optimal ratio was determined to be a five-fold excess of the Fc∆22 encoding plasmid, which was used for all further LV productions.

### Blinding the NiV glycoprotein G for its natural receptors

**Fig 2:**
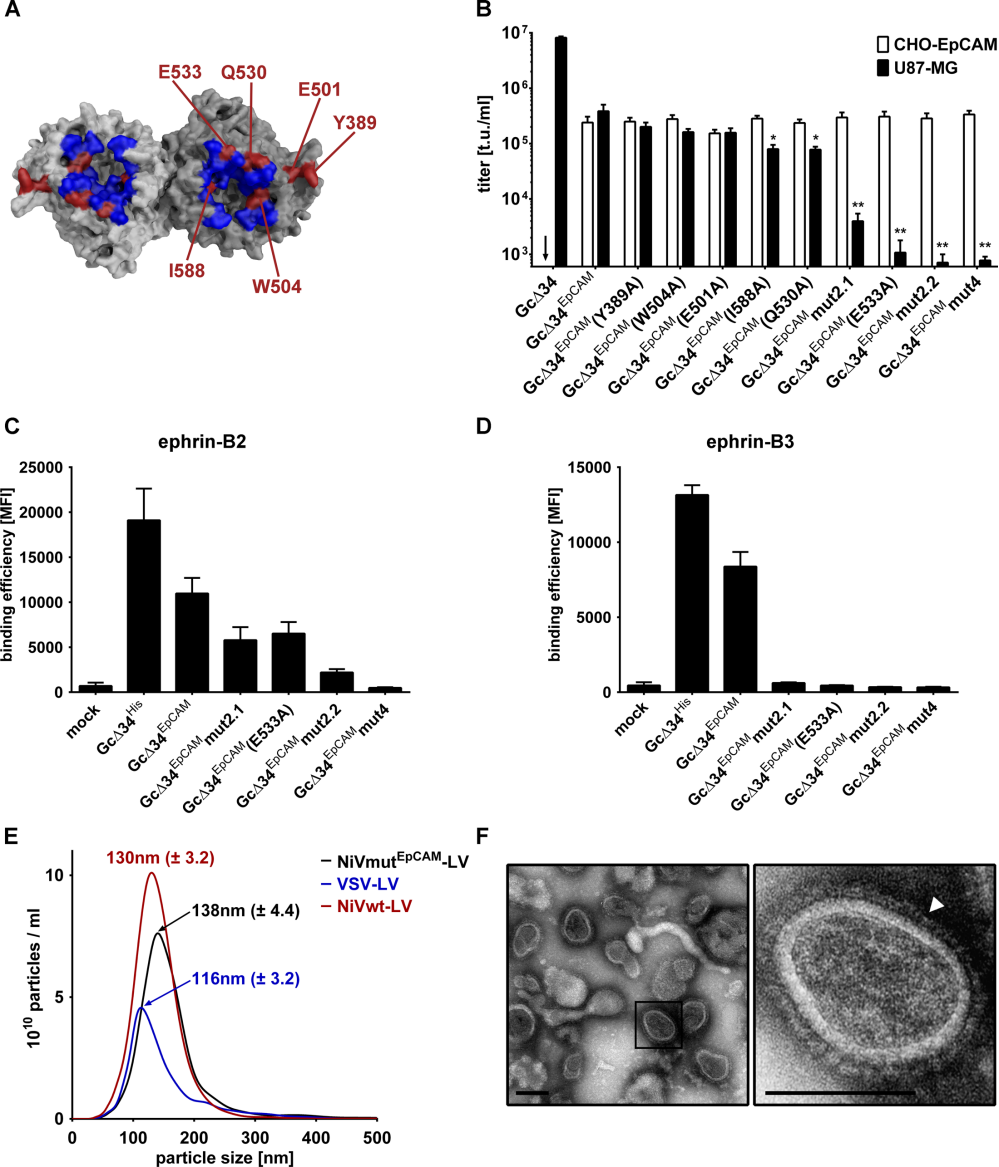
Mutation of the NiV glycoprotein to ablate natural receptor recognition. (**A**) Surface representation of top-view of NiV-G. G is shown as dimer by modeling its monomer crystal structure (Protein Data Bank (PDB) ID: 3D11) on the crystal structure of the Hendra virus G dimer (PDB ID: 2X9M) using PyMOL. The binding site for ephrin-B2 is depicted in blue [26]. Residues mutated in G to screen for their potential to ablate natural receptor tropism are shown in red. (**B**) Six different single mutations and the combinations E501A+W504A (Gc∆34^EpCAM^mut2.1), Q530A+E533A (Gc∆34^EpCAM^mut2.2), or E501A+W504A+Q530A+E533A (Gc∆34^EpCAM^mut4) were introduced into Gc∆34^EpCAM^. Unconcentrated vector stocks generated with the mutated G proteins were titrated on CHO-EpCAM (white bars; negative for natural NiV receptors) and U87-MG cells (black bars; positive for NiV receptors). NiV-LVs pseudotyped with Gc∆34/Fc∆22 having the natural NiV tropism (Gc∆34) served as control. Arrows indicate titers <6x10^2^ t.u./ml. Statistics refer to unmutated Gc∆34^EpCAM^. Titers of EpCAM-targeted LVs on CHO-EpCAM cells were not statistically different. (n = 4; mean ± standard deviations (SD) are shown; *, P<0.1 **, P<0.01 by one-way ANOVA with Dunnett's multiple comparisons test). (**C-D**) Binding of ephrin-B2 (**C**) and B3 (**D**) to NiV-G mutants is shown. HEK-293T cells were transfected either mock or with plasmids encoding the indicated G protein variants and then incubated with 1 μg/ml recombinant Fc-ephrin-B2 or -B3 prior to staining against the Fc-tag using FITC coupled anti-Fc antibody. The binding efficiencies of the different mutants to the receptors are shown as MFI (mean fluorescence intensity) values (n = 3; mean ± standard deviations (SD) are shown). (**E**) Concentrated vector stocks of Gc∆34^EpCAM^mut4/Fc∆22 pseudotyped LV vectors were generated and particle size was analyzed via single nanoparticle tracking analysis (NTA). Particle size measurement of one representative out of three independent stocks is shown (black). As control, concentrated vectors stocks of VSV-LV (blue) and NiVwt-LV (red) were analyzed. The mean size ± SD of the main peak out of three measurements of each particle type is indicated. (**F**) Electron microscopy of concentrated LV particles pseudotyped with Gc∆34^EpCAM^mut4/Fc∆22 proteins. The white arrowhead points to the NiV glycoproteins on the particle surface. Scale bar: 100 nm.

As the Gc∆34^EpCAM^ protein described above still recognizes the natural NiV receptors ephrin-B2 and B3, we aimed at completely restricting gene transfer to hEpCAM^+^ cells by destroying the natural receptor usage of NiV-G in the next step. For this purpose, six point mutations, previously described to influence the natural receptor recognition and cell entry of Nipah or Hendra virus [22–24], were introduced into Gc∆34^EpCAM^ individually or in combinations. All mutated residues localize in the contact area of NiV-G and ephrin-B2 (Fig 2A) [25,26]. For all Gc∆34^EpCAM^ proteins with a single point mutation the position and type of mutation is indicated in their respective designation. The three variants carrying combined mutations were termed as follows: Gc∆34^EpCAM^mut2.1 includes mutations E501A+W504A, Gc∆34^EpCAM^mut2.2 mutations Q530A+E533A, and Gc∆34^EpCAM^mut4 mutations E501A, W504A, Q530A and E533A. U87-MG cells, a glioblastoma cell line known to be highly susceptible for NiV infections, was used to determine ephrin-B2/B3 receptor usage by the LVs pseudotyped with the mutated Gc∆34^EpCAM^ proteins. To control that the mutations in Gc∆34^EpCAM^ did not affect the fusion-helper function of G but selectively inhibited recognition of ephrin-B2/B3, LVs were assessed for their ability to transduce ephrin-B2/B3 negative CHO-EpCAM cells. As expected, LVs pseudotyped with Gc∆34 and Fc∆22 transduced U87-MG cells but not CHO-EpCAM cells, while Gc∆34^EpCAM^ carrying LVs transduced both cell types (Fig 2B). Of the six single mutations three showed a significant negative impact on gene delivery into U87-MG cells (Fig 2B, black bars), with the mutation at position 533 (E533A) being most effective. Combining Q530A with E533A showed a slight advantage over the combination of E501A with W504A or the single mutations. None of the mutations significantly influenced the transduction of CHO-EpCAM cells (Fig 2B, white bars).

To clearly demonstrate that the mutations in Gc∆34^EpCAM^ interfere with binding to the natural NiV receptors, the ability to bind soluble recombinant ephrin-B2 and ephrin-B3 was analyzed by a flow cytometry-based binding assay. Interestingly, even though mutant E533A and Q530A+E533A (mut2.2) showed only very low levels of transduction, binding of ephrin-B2, but not of ephrin-B3, could still be detected (Fig 2C and 2D). Only binding of ephrin-B3 was clearly impaired indicating that LV entry into U87-MG is mediated by binding of NiV-G to ephrin-B3 (Fig 2D). Importantly, when combining the four mutations in construct Gc∆34^EpCAM^mut4, binding of recombinant ephrin-B2 dropped to background levels as well (Fig 2C). Therefore, this mutant was used for all further experiments to ensure entry and binding to be ephrin-B2/B3 independent.

To further characterize the NiVmut^EpCAM^-LV particles, their size was determined by single nanoparticle tracking (NanoSight). The average particle size peaked at 138 nm (± 4.4) (Fig 2E). This is a slight increase over NiVwt-LV (Gc∆34/Fc∆22 pseudotyped), which showed a peak size of 130 nm (± 3.2), probably due to fusion of the DARPin to the NiV-G protein. For comparison, VSV-LV, vector particles pseudotyped with the glycoprotein G of vesicular stomatitis virus, had a diameter of 116 nm (± 3.2) (Fig 2E). Electron microscopy of concentrated NiVmut^EpCAM^-LV stocks revealed numerous particles exhibiting the typical morphology of HIV-1 core particles. On their surface, a homogeneous high-density layer of spike proteins was readily detectable (Fig 2F).

**Fig 3:**
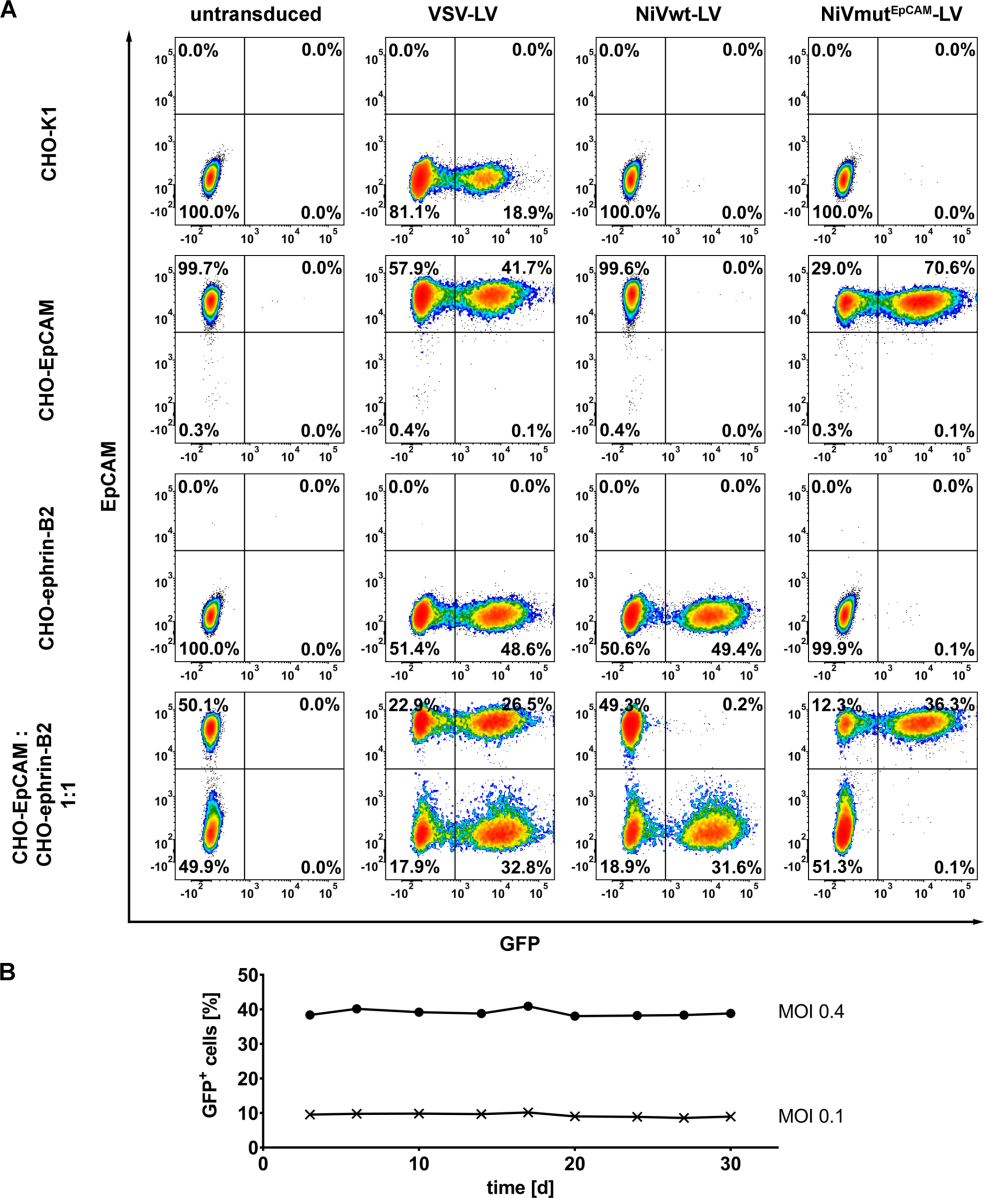
Selectivity of NiVmut^EpCAM^-LV for EpCAM^+^ cells. (**A**) Cell entry is EpCAM dependent but independent of ephrin-B2. Representative flow cytometry plots out of three independent experiments of CHO-K1, CHO-EpCAM, CHO-ephrin-B2 cells, and of a mixed culture composed of CHO-EpCAM and CHO-ephrin-B2 (1:1 ratio) monitored 72 h after transduction with NiVmut^EpCAM^-LV, NiVwt-LV or VSV-LV (MOI of 1). EpCAM expression was detected by an APC-coupled human EpCAM specific antibody. (**B**) To ascertain stability of transduction with the EpCAM-targeted vector, CHO-EpCAM cells were cultivated for further 30 days after transduction with the indicated MOIs. The percentage of GFP-positive cells was determined by flow cytometry at the indicated time points. One representative out of three independent experiments is shown.

To assess the selectivity of gene transfer mediated by NiVmut^EpCAM^-LV, CHO-K1 cells which are negative for both, EpCAM and ephrin-B2, CHO-ephrin-B2 and CHO-EpCAM cells were transduced either individually or in mixed cultures, where EpCAM^+^ and EpCAM^-^ cells are cultivated in close contact. VSV-LV transduced all these cell lines equally efficient. NiVwt-LV selectively transduced CHO-ephrin-B2 cells in single and mixed culture, while NiVmut^EpCAM^-LV selectively transduced only CHO-EpCAM cells (Fig 3A). On receptor-negative cells at most 0.1% GFP-positive cells were detectable. This was also the case for CHO-ephrin-B2 cells, demonstrating that natural receptor usage had been completely ablated by the introduced point mutations (Fig 3A). The selective gene transfer mediated by NiVmut^EpCAM^-LV was stable over a period of 30 days of cultivating the transduced cells (Fig 3B). GFP protein transfer can therefore be ruled out and integration of the *gfp* reporter gene into the host cell chromosomes assumed.

**Fig 4:**
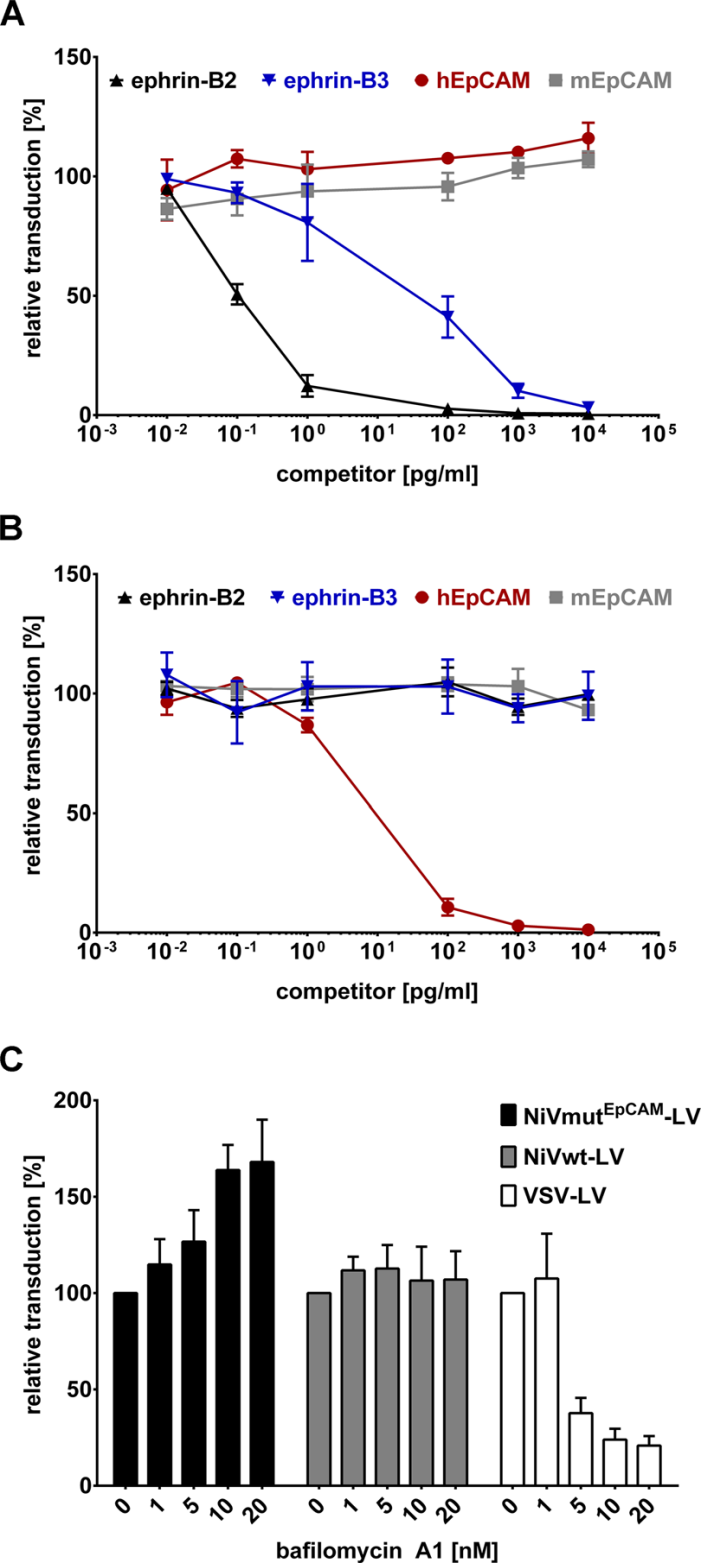
Receptor usage of NiVmut^EpCAM^-LV. To determine the receptor usage of NiVwt-LV (**A**) in comparison to NiVmut^EpCAM^-LV (**B**) a competition assay was performed by incubating the vector particles (MOI 0.4) for 1 h at 4°C with increasing amounts of the entire extracellular domain of human ephrin-B2 (black lines), ephrin-B3 (blue lines), human EpCAM (red lines) or murine EpCAM (grey lines). Following incubation, cells were transduced and analyzed for GFP expression by flow cytometry 72 h post transduction. Data are normalized to transduction efficiency measured without pre-incubation with recombinant protein (n = 3). (**C**) CHO-EpCAM and CHO-ephrin-B2 cells (1x10^4^) were pre-treated with increasing amounts of bafilomycin A1 for 30 minutes prior to transduction of cells with NiVmut^EpCAM^-LV and NiVwt-LV at an MOI of 0.4. VSV-LV served as a control as cell entry of this vector is described to be pH-dependent. Relative transduction rates compared to cells transduced in absence of bafilomycin A1 were determined by flow cytometry 72 h post transduction (n = 3).

To verify cell entry via the targeted receptor, NiVwt-LV or NiVmut^EpCAM^-LV were incubated with increasing amounts of the entire extracellular domain of human ephrin-B2, ephrin-B3, human EpCAM or murine EpCAM as a control at 4°C for 1 h prior to transduction of target cells. For NiVwt-LV, a dose-dependent decrease in the transduction of CHO-ephrin-B2 cells with increasing concentrations of recombinant ephrin-B2 was documented, whereas human or murine EpCAM did not influence transduction (Fig 4A). Ephrin-B3, which is known to bind to the same site on the NiV G protein as ephrin-B2, also competed with gene transfer via ephrin-B2 but at much higher concentrations. NiVmut^EpCAM^-LV, in contrast, was only competed by human EpCAM but neither by murine EpCAM nor ephrin-B2 or -B3 (Fig 4B).

To examine if cell entry of the NiV-pseudotyped LVs was pH-independent, as described for Nipah virus [27], acidification of endosomes was blocked with bafilomycin A1. VSV-LV mediated gene transfer was strongly reduced by bafilomycin A1 which corresponds to its well established pH-dependent cell entry [28] (Fig 4C). In contrast, the transduction by NiVwt-LV was not affected. Surprisingly, gene transfer mediated by NiVmut^EpCAM^-LV was even enhanced upon bafilomycin A1 treatment (Fig 4C). These data demonstrate that NiVmut^EpCAM^-LV enters cells pH-independently.

### Expanding the system to additional target receptors

**Fig 5:**
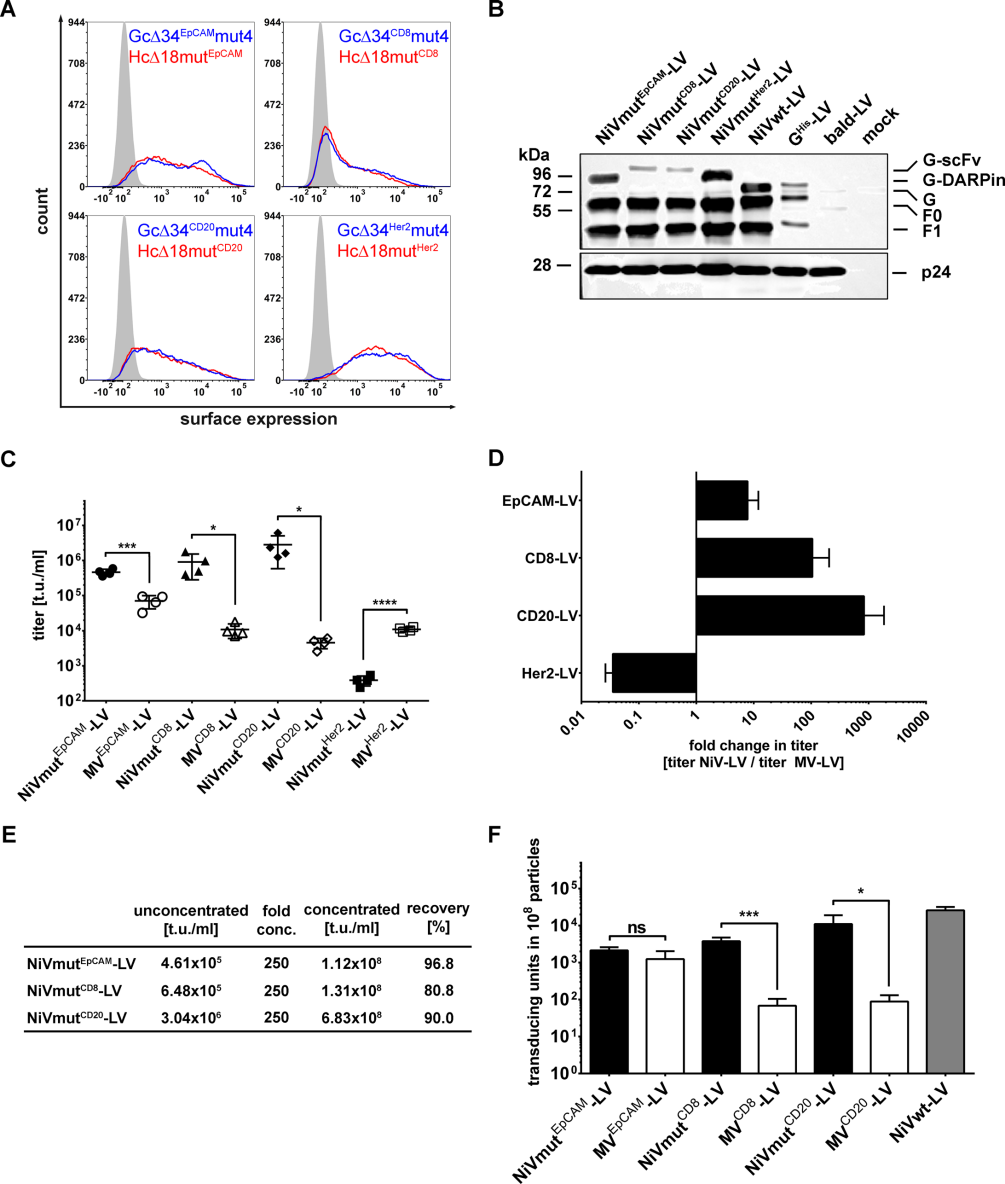
Expanding the system to additional target receptors. (**A**) Surface expression of NiV G proteins (blue line) targeted to four different receptors was compared to that of the corresponding MV H protein counterparts (red line). All expression plasmids encoding the different constructs were transfected into HEK-293T cells. Surface expression was analyzed after 48 hours using a His-tag-specific antibody (PE-labeled). Mock transfected cells (filled curves) served as negative control. One representative out of three experiments is shown. For quantitative data see S3 Fig. (**B**) Western blot analysis of NiVmut^EpCAM^-LV, NiVmut^CD8^-LV, NiVmut^CD20^-LV, and NiVmut^Her2^-LV. For generation of the vectors used for Western blot analysis, F protein C-terminally tagged with the AU1 immunological tag was used to allow detection via the anti-AU1 antibody. Incorporation of the G variants was detected via an anti-His antibody. 2.5x10^10^ particles per sample were used. Mock transfected cells (mock) as well as bald particles without glycoproteins (bald-LV) served as controls. In addition, particles pseudotyped with full-length His-tagged G and AU1 tagged F (G^His^-LV) as well as particles pseudotyped with Gc∆34^His^/Fc∆22-AU1 (NiVwt-LV) were used. For quantitative data see S4 Fig. (**C**) Titers of receptor-targeted NiV-LVs and their MV-LV counterparts. Unconcentrated stocks of EpCAM-targeted vectors were titrated on CHO-EpCAM cells, CD20-targeted vectors on Raji, CD8 targeted-vectors on Molt4.8 and Her2/*neu*-targeted vectors on SK-OV-3 cells. (n = 4; mean ± standard deviations (SD) are shown; *, P<0.1; ***, P<0.001; ****, P<0.0001 by unpaired *t-*test). (**D**) Fold change in titers (t.u./ml) determined by normalizing the titers of NiV glycoprotein based LVs to those of the corresponding MV glycoprotein based LVs. (**E**) Concentration of vector stocks from (**C**) by centrifugation. (**F**) Number of transducing units per 10^8^ physical particles of NiV and MV glycoprotein based LVs. Particle numbers were determined by single nanoparticle tracking analysis (NTA) (n = 4; mean ± standard deviations (SD) are shown; *, P<0.1; ***, P<0.001; ns, not significant by unpaired *t-*test).

Next, we asked if other surface proteins can be targeted by engineered NiV glycoproteins as well. Human CD8, a marker for cytotoxic T cells, human CD20, a marker for B cells, and Her2/*neu*, a marker for breast cancer, were targeted as described previously for MV glycoprotein pseudotyped LVs [29–31]. As targeting ligands, we used DARPin 9.29 for Her2/*neu *[30], and single chain antibodies (scFv) specific for CD8 [29] or CD20 [31]. Each G variant was expressed at the cell surface to the same level as that of the corresponding MV-HcΔ18mut variant, respectively (Fig 5A; S3 Fig). In addition, they were incorporated into the LV particles with a tendency for higher incorporation levels of the DARPin- over the scFv-displaying G proteins (Fig 5B; S4 Fig). Several batches of all four NiV-based and the corresponding MV-based receptor-targeted vectors were generated and titrated on cell lines expressing the targeted receptor. Interestingly, we observed 10-100-fold higher titers for NiVmut^EpCAM^-LV, NiVmut^CD20^-LV, and NiVmut^CD8^-LV compared to their MV-based counterparts (Fig 5C and 5D). Notably, NiVmut^Her2^-LV was an exception. Here, the titer was reduced 30-fold when compared to its MV-based counterpart (Fig 5C and 5D). Importantly, concentrating the vector particles did not impair transduction efficiency with recovery rates of 80.8 to 96.9% (Fig 5E). When supernatant from packaging cells cultivated in ten T175 flasks was concentrated down to 0.6 ml, titers of above 10^8^ t.u./ml were obtained.

To identify potential reasons for the increased titers of receptor-targeted LVs based on the NiV glycoproteins, particle numbers in all concentrated vector stocks were determined by single nanoparticle tracking analysis. All NiV-based LV stocks contained between three- to five-fold higher particle numbers than the corresponding MV-based LV stocks (S5 Fig). In 10^8^ particles, all the NiV pseudotyped LV stocks contained more transducing units, than the corresponding MV-based LVs (Fig 5F). This holds true especially for the NiV-based CD8 and CD20 targeted LVs which exhibited more than 10-fold higher values (Fig 5F). Thus, the higher gene transfer activity observed for the NiV-based receptor-targeted LVs must be due to a mixture of two aspects: On the one hand, more particles are released during production and on the other hand, the particles are more active.

### NiV-based lentiviral vectors are resistant towards intravenous immunoglobulins

**Fig 6:**
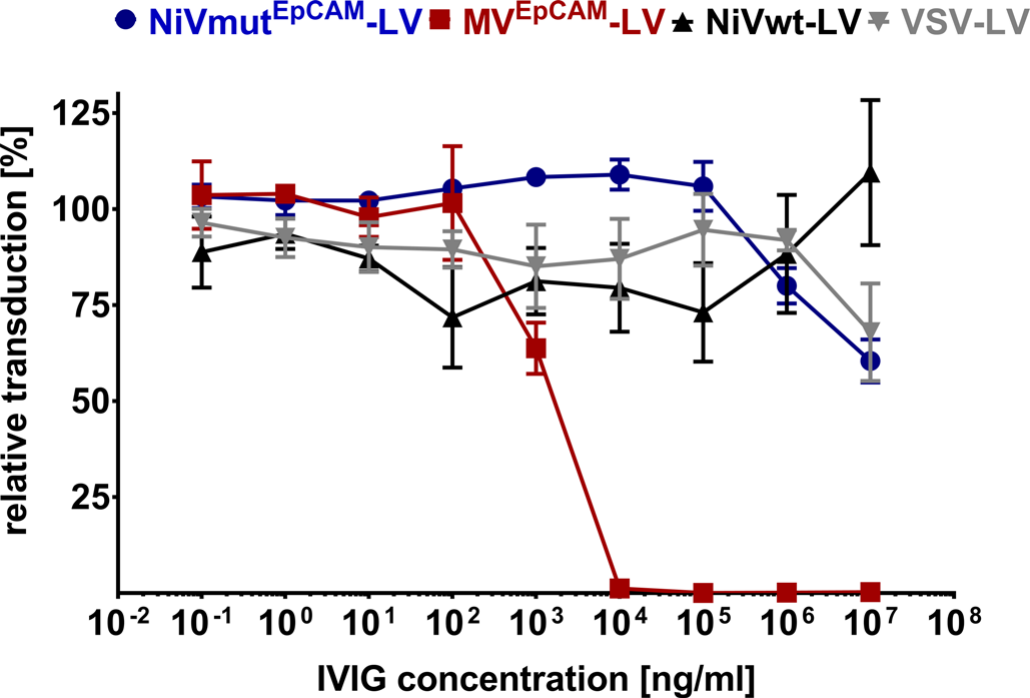
Neutralization of NiV glycoprotein pseudotyped LVs. CHO-EpCAM or CHO-ephrin-B2 cells were transduced with NiVmut^EpCAM^-LV (blue), MV^EpCAM^-LV (red), NiVwt-LV (black), or VSV-LV (grey) at an MOI of 0.4 after incubation with serial dilutions of pooled human serum (IVIG) for 2 h at 37°C. After 72 h, GFP^+^ cells were determined by flow cytometry. The number of GFP^+^ cells relative to the untreated control is shown (n = 3).

Due to the vaccination against measles virus, MV-based LVs become neutralized by human serum to at least some extent even though receptor-targeted MV-LVs exhibit some level of protection [32]. Since there is no vaccination against NiV and the outbreaks were limited to a few cases in Malaysia, Bangladesh and India [33], there should be no neutralizing antibodies present in humans. To cover the widest range of human serum donors, intravenous immunoglobulin (IVIG; Intratect), which contains serum from many thousand donors, was incubated with NiVwt-LV, NiVmut^EpCAM^-LV, MV^EpCAM^-LV and VSV-LV at increasing concentrations prior to the transduction of target cells. MV^EpCAM^-LV showed a dose-dependent decrease in transduction rates and was completely neutralized at 10 μg/ml of IVIG. In contrast, VSV-LV, NiVwt-LV and also NiVmut^EpCAM^-LV were resistant against IVIG at all concentrations used and must thus be at least 10,000-fold less sensitive against human immunoglobulin than the corresponding MV-based vector (Fig 6). These results suggest that receptor-targeted vectors based on NiV glycoproteins will not be neutralized when injected into humans.

### NiVmut^CD8^-LV selectively transduces CD8^+^ T cells in human PBMC

**Fig 7:**
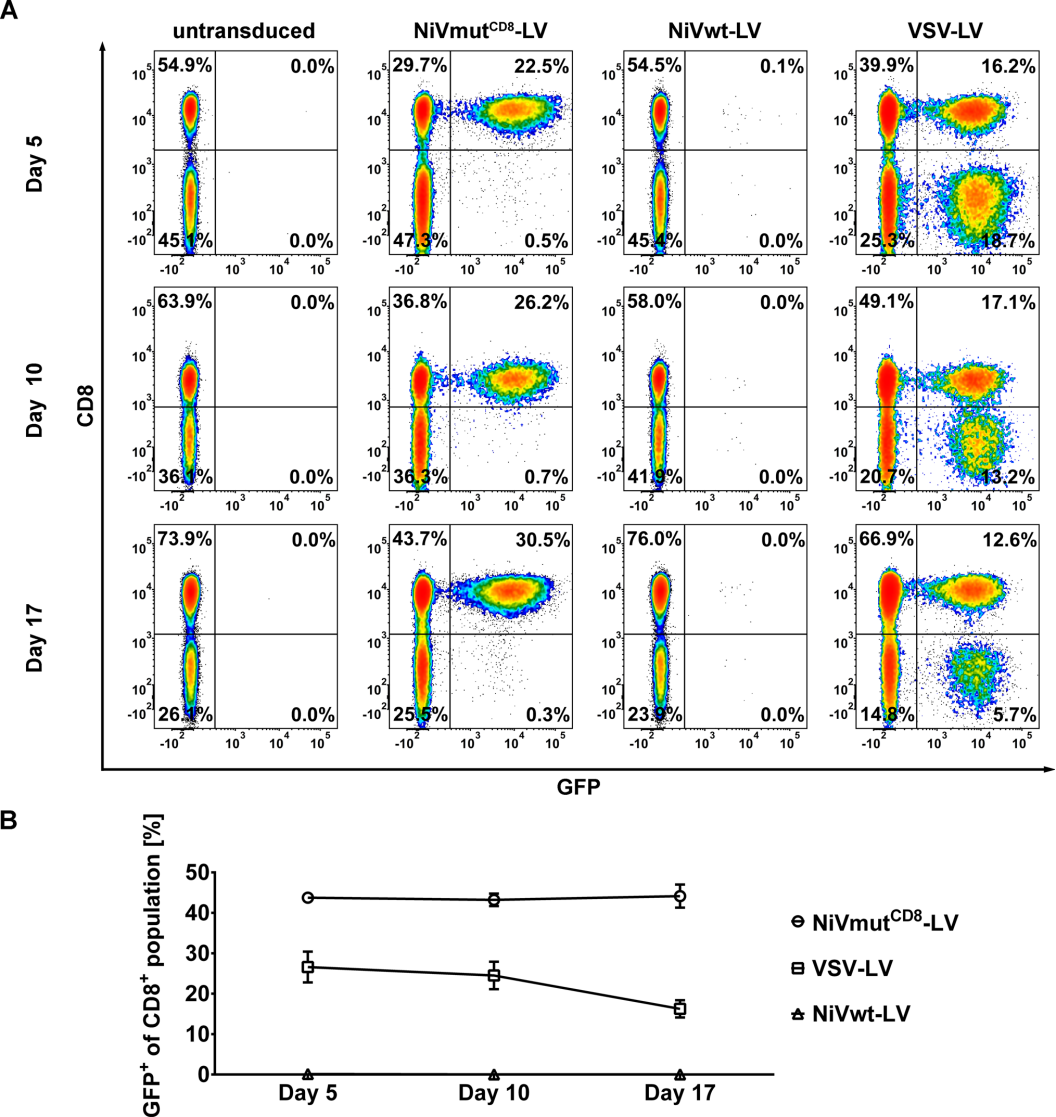
Selective transduction of CD8^+^ human PBMC. (**A**) Freshly isolated human PBMC were activated for three days and then transduced with NiVmut^CD8^-LV, NiVwt-LV or VSV-LV at an MOI of 2, respectively. Representative flow cytometry plots out of three independent experiments are shown. GFP fluorescence was measured via flow cytometry at day 5, 10 and 17 after transduction. CD8^+^ cells were stained with APC coupled anti-CD8 antibody. (**B**) Percentage of GFP positive cells within the CD8^+^ population was measured at day 5, 10 and 17 after transduction (n = 3).

To prove that the established system does not only show selective transduction in cell lines but does also transfer genes into primary cells, human peripheral blood mononuclear cells (PBMC) were chosen as targets. Freshly isolated PBMC were activated for three days and transduced with NiVmut^CD8^-LV. As controls, cells were transduced with VSV-LV or NiVwt-LV. GFP expression was followed over a period of 5, 10 and 17 days. VSV-LV transduced both cell fractions, CD8^+^ and CD8^-^ (Fig 7A, top right diagram). NiVwt-LV was unable to transduce any cell type present in human PBMC (Fig 7A). NiVmut^CD8^-LV, in contrast, selectively transduced the CD8^+^ cells at high efficiency. The gene transfer mediated by NiVmut^CD8^-LV into CD8^+^ cells was stable for at least 17 days (Fig 7B). Notably, PBMC transduced by VSV-LV showed a significant decrease in GFP^+^ cells over this period. This was most likely due to cells that had not integrated the GFP gene but taken up GFP as protein. Thus, NiVmut^CD8^-LV cannot only transduce selectively CD8^+^ populations in human PBMC but also ensures stable gene expression over time.

### Characterization of NiVmut^Her2^–LV

**Fig 8:**
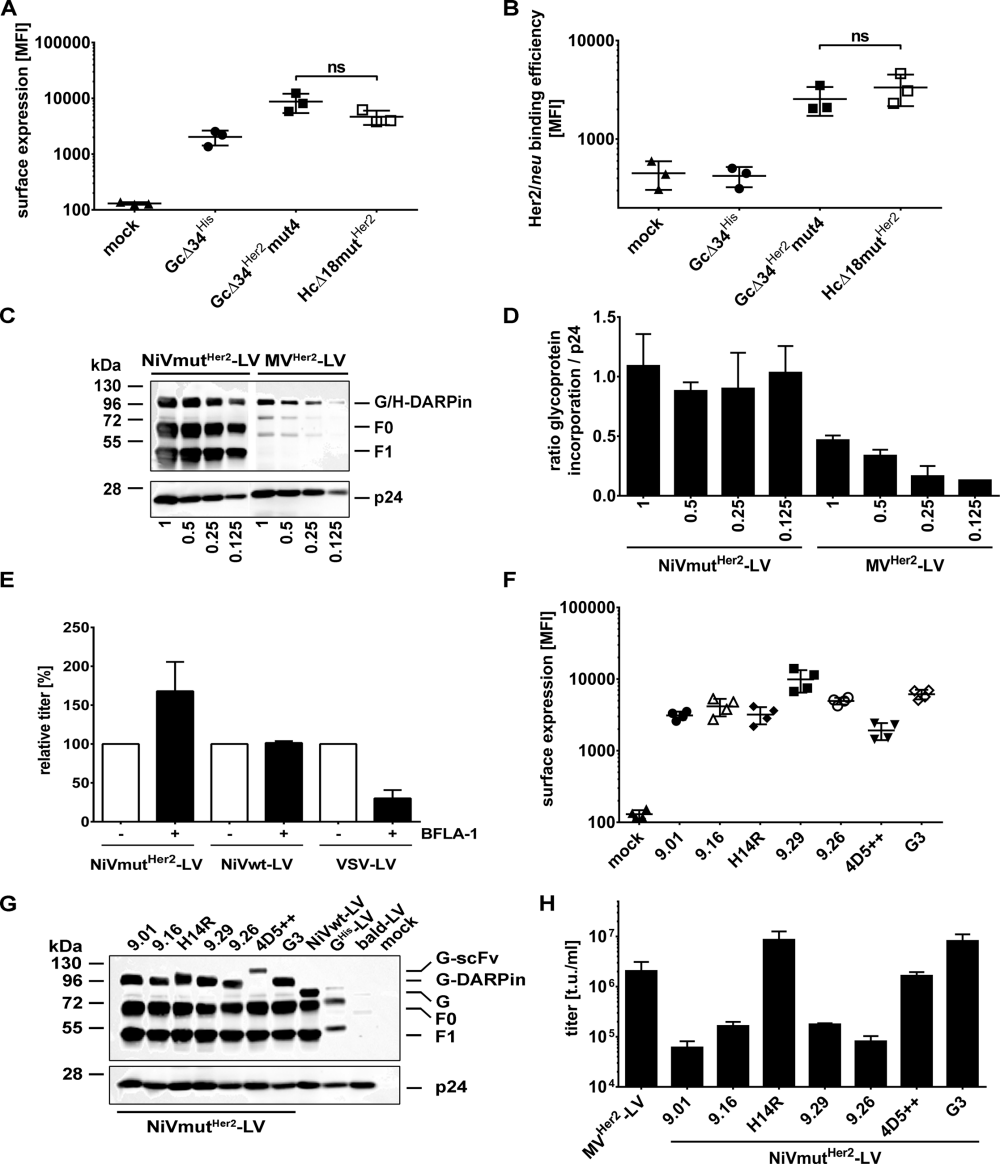
Characterization of NiVmut^Her2^-LV. (**A**) Surface expression of Gc∆34mut^Her2^ and Hc∆18mut^Her2^ in comparison to Gc∆34^His^ on HEK-293T cells transiently transfected with plasmids encoding the different glycoproteins compared to mock transfected cells as determined by flow cytometry. Cells were stained with PE coupled anti-His antibody. Mean fluorescence intensities of three independent measurements are shown (n = 3; mean ± standard deviations (SD) are shown; ns, not significant by unpaired *t-*test). See S6 Fig for exemplary raw data. (**B**) Binding of recombinant Her2/*neu* to the engineered glycoproteins. Gc∆34^Her2^mut4, Hc∆18mut^Her2^ and Gc∆34^His^ were expressed in HEK-293T cells, incubated for 1 h at 4°C with 1 µg/ml recombinant Fc-Her2/*neu* prior to staining against the Fc-tag using FITC coupled anti-Fc antibody. Mean fluorescence intensities of three experiments are shown (n = 3; mean ± standard deviations (SD); ns, not significant by unpaired *t-*test). See S6 Fig for exemplary raw data. (**C**) Western blot analysis of NiV- and MV-based Her2/*neu* targeted LVs. 2.5x10^10^ (1), 1.25x10^10^ (0.5), 6.25x10^9^ (0.25) and 3.125x10^9^ (0.125) particles were applied per sample, respectively. The glycoproteins G and H were detected by anti-His antibody. The NiV F glycoprotein was detected via the AU1-tag specific antibody. One representative out of three Western blots is shown. The central lane between the NiV and MV samples containing the molecular weight marker was removed. (**D**) Incorporation levels of Gc∆34^Her2^mut4 and Hc∆18mut^Her2^. Three independently generated stocks of NiVmut^Her2^-LV and MV^Her2^-LV were subjected to Western blot analysis applying four different particle numbers as shown in (**C**). Average chemiluminescence values for the glycoproteins G and H were then normalized to those of p24 (n = 3 for all NiVmut^Her2^-LV dilutions and MV^Her2^-LV dilutions 1 and 0.5; n = 2 for dilutions 0.25 and n = 1 for dilution 0.125 of MV^Her2^-LV; mean ± standard error of the mean (SEM) are shown). (**E**) Bafilomycin A1 sensitivity of NiVmut^Her2^-LV. NiVmut^Her2^-LV, NiVwt-LV and VSV-LV were titrated on SK-OV-3 cells in presence or absence of 20 nM bafilomycin A1. Relative titers of vectors in presence of bafilomycin A1 to untreated control are shown (n = 3). (**F**) Exchanging the DARPin 9.29 with alternative Her2/*neu*-specific targeting domains. Mean fluorescence intensities of surface expression of Gc∆34^Her2^mut4 variants in which DARPin 9.29 was replaced by DARPins 9.01, 9.16, 9.26, H14R, G3 or the scFv 4D5++ after transient transfection of HEK-293T cells with the corresponding expression plasmids compared to mock transfected cells as determined by flow cytometry. Cells were stained with PE-coupled anti-His antibody (n = 4; mean ± standard deviations (SD) are shown). Examples of representative flow cytometry plots are shown in S7 Fig. (**G**) Western blot analysis of the different NiV glycoprotein based vectors targeted to Her2/*neu*. The G variants were detected via an anti-His antibody, and F by AU1-tag specific antibodies. 2.5x10^10^ particles per sample were applied. Mock transfected cells (mock) as well as bald particles without glycoproteins (bald-LV) served as controls. In addition, particles pseudotyped with full-length His-tagged G and AU1 tagged F (G^His^-LV) as well as particles pseudotyped with Gc∆34^His^/Fc∆22-AU1 (NiVwt-LV) were used. For quantitative data see S8 Fig. (**H**) Titers of concentrated vector stocks of the different Her2/*neu* specific NiV-LVs and of MV^Her2^-LV as determined on SK-OV-3 cells (n = 3; mean ± standard deviations (SD) are shown).

To investigate the underlying mechanism resulting in the reduced titers of Her2/*neu*-targeted NiV-based LV, surface expression of the Gc∆34^Her2^mut4 protein on HEK-293T producer cells was assessed first, as this is a critical step for incorporation into budding vector particles. Surface expression levels of Gc∆34^Her2^mut4 and the corresponding MV-derived Hc∆18mut^Her2^ did not differ and were slightly enhanced compared to Gc∆34^His^, which is most likely due to a better recognition of the His-tag when displayed on top of the DARPin (Fig 8A). Next, the ability of Gc∆34mut^Her2^mut4 to bind recombinant Her2/*neu* was investigated. For this purpose, HEK-293T cells transfected with plasmids encoding Gc∆34^Her2^mut4, Hc∆18mut^Her2^, or Gc∆34^His^ were incubated with Her2/*neu* and subsequently stained for Her2/*neu* binding. As expected, Gc∆34^His^ and mock transfected cells showed no binding of Her2/*neu*. In contrast, the NiV- and MV-based Her2/*neu*-specific constructs showed an identical binding efficiency of recombinant Her2/*neu* (Fig 8B).

To quantitatively compare the amounts of Gc∆34^Her2^mut4 and Hc∆18mut^Her2^ in LV particles, we applied different amounts of particles from several independently generated batches of vector stocks to Western blot analysis making use of the His-tag in both proteins for detection. There were reproducibly higher amounts of G than of H protein present in each batch and dilution analyzed (Fig 8C and 8D). At the highest dilution, H was detectable in only one sample. On the average of all dilutions and vector batches analyzed, there was 3.14 ± 0.66 (n = 8) fold more Gc∆34^Her2^mut4 than Hc∆18mut^Her2^ incorporated. Since Gc∆34^Her2^mut4 was able to bind Her2/*neu* and was incorporated into LV particles at even higher levels than Hc∆18mut^Her2^, we tested next, if the vector particles had lost their gene transfer activity due to an increased endocytosis and subsequent degradation by endo-lysosomal proteases. For this purpose, endosomal acidification of SK-OV-3 cells (positive for Her2/*neu* and ephrin-B3) was blocked by bafilomycin A1. As shown before, transduction by VSV-LV was inhibited whereas transduction by NiVwt-LV was not influenced (Fig 8E). Interestingly, gene transfer by NiVmut^Her2^-LV was rather enhanced by bafilomycin A1 by a factor similar to that seen for NiVmut^EpCAM^-LV (Figs 8E and 4C). Thus, the defect in transduction of the Her2/*neu* specific NiV-LV was likely not caused by loss of particles to acidified endosomal compartments.

DARPin 9.29 had been used in NiVmut^Her2^-LV as this targeting domain was found to be the best for the MV-glycoprotein-based system [30]. To test, if the targeting domain may make a difference, DARPin 9.29 was exchanged against five different Her2/*neu* specific DARPins (9.01, 9.16, 9.26, H14R, G3) [34,35] and one trastuzumab-derived scFv (4D5++) [36]. All Gc∆34mut4 fusion proteins were expressed on the surface of HEK-293T producer cells, with a tendency for the 9.29 DARPin to mediate higher and the scFv 4D5++ to mediate lower expression levels (Fig 8F). Particle incorporation levels for most of the new G variants were in the same range as that for the 9.29 displaying Gc∆34mut4, with the only exception for the scFv 4D5++ (Fig 8G; S8 Fig). Notably, DARPins H14R and G3 as well as the scFv 4D5++ mediated gene transfer activities comparable to or even higher than that of MV^Her2^-LV (Fig 8H). Enhanced binding to Her2/*neu* could be excluded as being causative, since Gc∆34mut4 proteins fused to these targeting ligands were similar or rather less efficient in binding Her2/*neu* (S9 Fig). Remarkably, the two DARPins as well as the scFv bind to domain IV of Her2/*neu*, the most membrane-proximal domain, whereas the four other DARPins, which resulted in low functional titers, bind to domain I [34,35,37].

**Fig 9:**
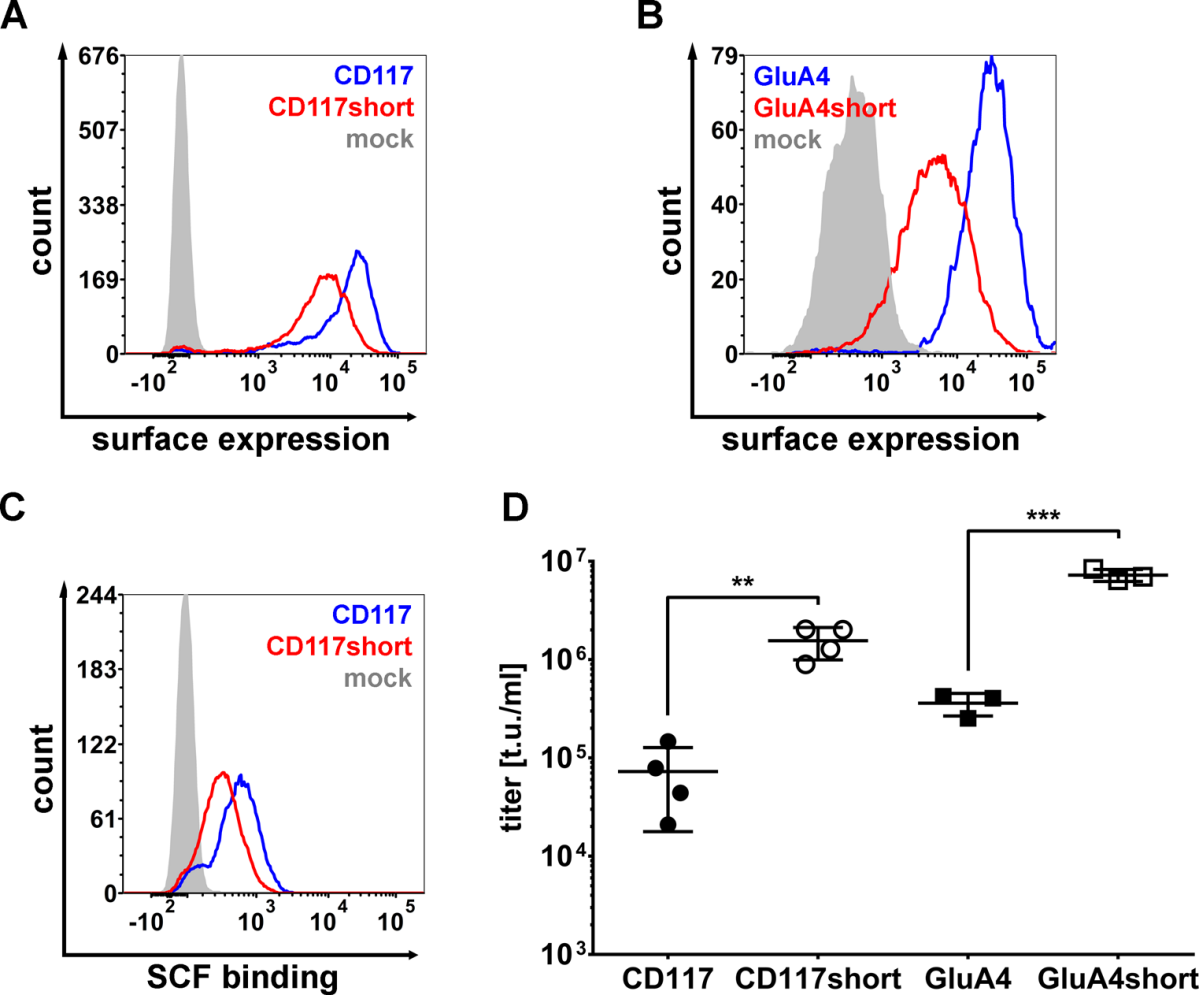
CD117- and GluA4-targeted NiV-LVs. (**A**) Surface expression of CD117 (blue line) and CD117short (red line) on stably expressing HT1080 cells compared to the parental cell line (filled curve) as determined by flow cytometry. Cells were stained with PE-coupled CD117 antibody. One representative out of four experiments is shown. See S10 Fig for quantitative data. (**B**) Surface expression of GluA4 (blue line) and GluA4short (red line) on stably expressing HT1080 cells compared to the parental cell line (filled curve) as determined by flow cytometry. Cells were stained with PE-coupled myc-tag antibody. One representative out of three experiments is shown. See S10 Fig for quantitative data. (**C**) Binding of recombinant SCF to CD117 and CD117short. Fc-SCF was produced in HEK-293T cells by transient transfection. HT1080, HT1080-CD117 and HT1080-CD117short were incubated for 1 h at 4°C with the same volumes of recombinant Fc-SCF prior to staining against Fc-tag using FITC coupled anti-Fc antibody. One representative out of three experiments is shown. See S10 Fig for quantitative data. (**D**) Titers of concentrated stocks of NiVmut^CD117^-LV and NiVmut^GluA4^-LV. NiVmut^CD117^-LV was titrated on HT1080-CD117 and HT1080-CD117short cells (n = 4; mean ± standard deviations (SD) are shown; **, P<0.01by unpaired *t-*test). NiVmut^GluA4^-LV was titrated on HT1080-GluA4 and HT1080-GluA4short cells (n = 3; mean ± standard deviations (SD) are shown; ***, P<0.001 by unpaired *t-*test).

To further investigate a potential preference of the NiV glycoproteins for membrane-proximal binding sites, we targeted NiV-LV particles to two further receptors with large extracellular parts, the human c-kit receptor (CD117) and the glutamate receptor 4 (GluA4). CD117 was targeted by displaying its natural ligand stem cell factor (SCF), and GluA4 by a recently selected DARPin, in each case via a (G_4_S)_3_ linker on Gc∆34mut4. CD117 is a tyrosine kinase receptor composed of five Ig-like domains of which the first two domains supported by domain III form the SCF binding site [38]. The crystallized extracellular part of CD117 forms a rigid structure which projects the SCF binding site away from the cell membrane by about 120 Å [38,39]. GluA4 is a typical channel protein with an amino-terminal domain (ATD) reaching up to 120 Å away from the cell membrane. As alternative receptors, we moved domains I-III of CD117 by about 70 Å and the ATD of GluA4 by about 50 Å closer to the cell membrane by fusing them to the CD4 transmembrane domain, respectively. All receptors were stably expressed in human fibrosarcoma HT1080 cells. The Gc∆34mut4 variants as well as the receptors were readily detected at the cell surface by flow cytometry with a tendency for the shortened receptors to reach lower surface expression levels (Fig 9A and 9B; S10 Fig). For CD117 this was verified by using recombinant SCF instead of the CD117-specific antibody to detect surface expression (Fig 9C; S10 Fig). The titers of NiVmut^CD117^-LV and of NiVmut^GluA4^-LV particles were in a similar low range as that of NiVmut^Her2^-LV when added to cells expressing the unmodified receptors (Fig 9D). Titers increased by at least 20-fold on cells expressing the shortened receptors (Fig 9D), thus supporting the idea of more efficient cell entry via membrane proximal receptors.

## Discussion

Here we describe successful engineering of the NiV glycoproteins for LV pseudotyping and receptor targeting, which allowed us to rapidly generate a large series of glycoprotein variants attaching to a variety of cell surface proteins and assessing cell entry. For pseudotyping, distinct truncations in G (Gc∆33 and Gc∆34) and F protein (Fc∆22) were found to be optimal. Our data are thus in line with those of Witting et al (2013) for G protein, and with those of Palomares et al. (2012) for F protein. For both, F and G, the enhanced titers correlated well to an enhanced incorporation into LV particles, suggesting steric hindrance as likely explanation for the need for cytoplasmic tail truncations.

In the second engineering step we eliminated use of the natural NiV receptors ephrin-B2 and ephrin-B3 by introducing point mutations into the Gc∆34^EpCAM^ protein. For identifying the most effective mutations, we relied on the G protein structure and previously identified contact residues [22,23,25,26]. Yet, this turned out to be challenging, since the picomolar affinity of G for ephrin-B2 is among the strongest viral envelope-receptor interactions known [40,41]. Accordingly, we found that mutations E533A and W504A, the previously identified key residues for receptor attachment [22,23], were not sufficient to destroy ephrin-B2 binding completely, either individually or in combination (E533A/Q530A and E501A/W504A). However, combining both double mutations ultimately diminished binding to a level below detection. Importantly, transduction via the targeted EpCAM receptor was unimpaired by these mutations. Off-target transduction tested in CHO cells overexpressing ephrin-B2 was barely detectable and at least 1000-fold reduced when compared to the transduction of CHO cells overexpressing EpCAM. Since ephrin-B2 is widely expressed in the organism, including microvascular endothelial cells [42], having achieved complete abrogation of LV particle attachment to ephrin-B2 is an important step towards efficient *in vivo* gene delivery with receptor-targeted LVs.

NiV and MV enter cells by pH-independent membrane fusion at the cell membrane. EpCAM is known to be rapidly internalized upon antibody binding [21,43]. It is therefore likely that also binding of EpCAM-targeted vector particles induces internalization of EpCAM together with the bound particle. Interestingly, bafilomycin A1 enhanced gene delivery by NiVmut^EpCAM^-LV but, as expected, substantially reduced that mediated by VSV-LV, which is known to rely on pH-dependent entry [28]. Bafilomycin A1 is a selective inhibitor of the V-ATPase preventing the influx of protons into endosomes [44,45]. Thus, in the presence of bafilomycin A1 endocytosed NiVmut^EpCAM^-LV particles are less degraded by pH-dependent endo-/lysosomal proteases and can therefore enter the cytoplasm via fusion of the LV envelope with the endosomal membrane more efficiently [46]. Notably, a similar observation has been made for Her2/*neu* targeted MV-LVs using chloroquine as inhibitor [47]. Although being more unspecific, chloroquine also neutralizes the low pH in endosomes. It is well conceivable that in both settings more particles can escape the endosomes by membrane fusion and then contribute to the observed enhanced gene delivery rates.

**Fig 10:**
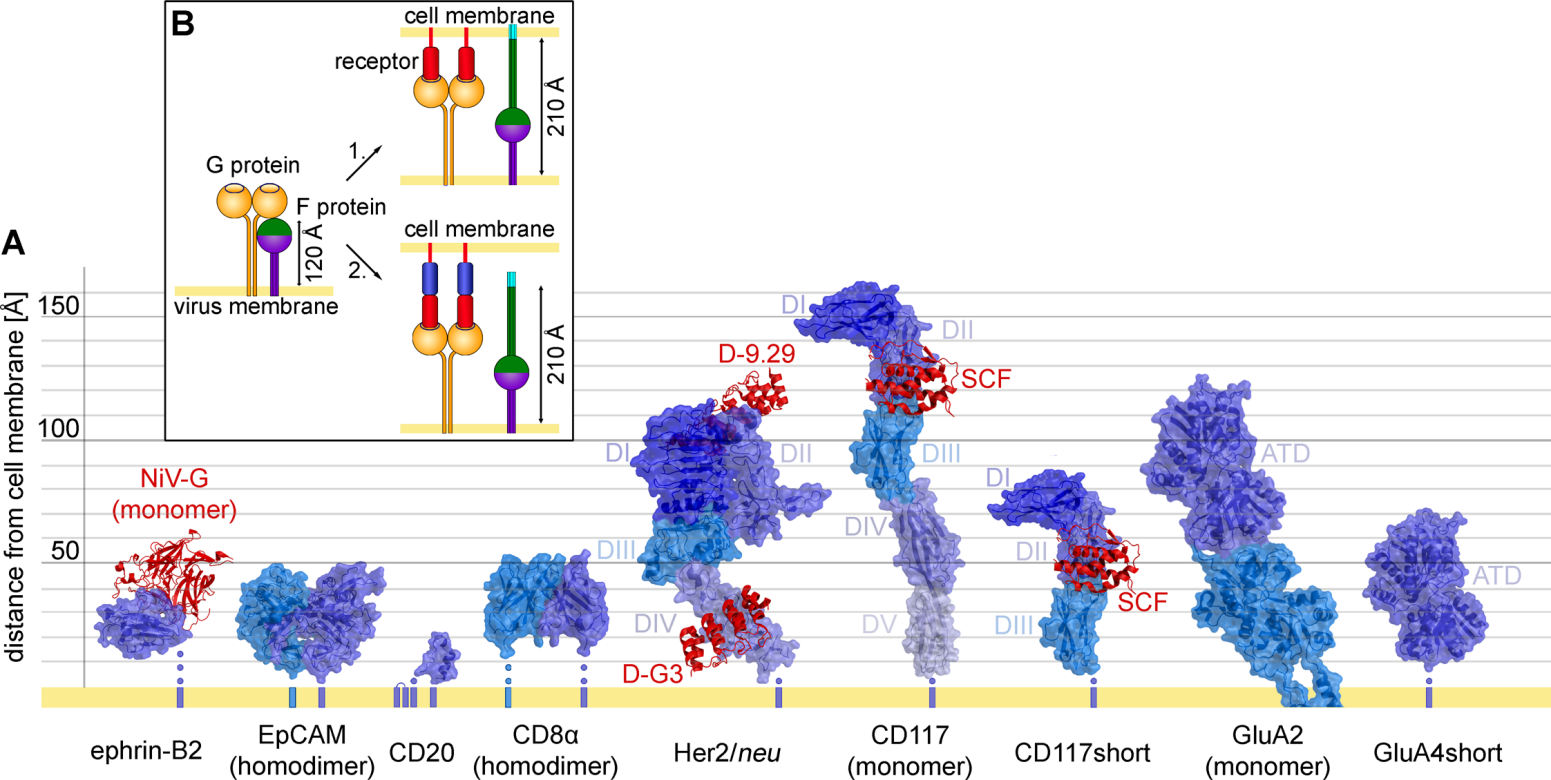
Position of the binding site on the targeted receptor determines cell entry. (**A**) Three-dimensional structures of the targeted receptors and the positions of their binding sites relative to the cell membrane. Surface representation of the extracellular domains of ephrin-B2 (PDB ID: 2VSM), EpCAM (PDB ID: 4MZV), CD20 (PDB ID: 3PP4), CD8 (PDB ID: 1CD8), Her2/*neu* (PDB ID: 1N8Z), CD117 (PDB ID: 2E9W), CD117short (adapted from PDB ID: 2E9W). For GluA4 the crystal structure of the closely related GluA2 including the transmembrane domain is shown (PDB ID: 3KG2). For GluA4short, the amino terminal domain (ATD) of GluA4 (PDB ID: 4GPA) is shown. Non-crystalized membrane-proximal amino acids (aa) of undefined structure are indicated with blue dots, each dot representing about 20 residues (ephrinB2: 64 aa; CD20: 25 aa (the structure of the small 6 aa loop is not available either); CD8α: 47 aa; Her2/*neu*: 23 aa; CD117: 17 aa; CD117short: 24 aa, GluA4short: 24 aa). When available, the structure of the complex between the target receptor and the targeting domain (red) is shown: Her2/*neu* with bound DARPin-9.29 (D-9.29, PDB ID: 4HRL) and DARPin-G3 (D-G3, PDB ID: 4HRN) (adapted from [37]). The CD117 ligand, SCF, is shown bound to CD117 and CD117short. For the natural NiV receptor ephrin-B2 the complex with the bound NiV-G monomer is shown. (**B**) Molecular model for the distance effect and its implication for NiV-mediated membrane fusion. In absence of receptor binding, F is in its prefusion state with the fusion peptide (light blue) being covered within the globular head (left). Upon attachment of G to its cell surface receptor, conformational changes are induced resulting in the projection of the fusion peptide followed by its insertion into the cell membrane (top right). If the attached binding site on the receptor is too far away from the cell membrane, the fusion peptide cannot insert and cell entry will not proceed (bottom right). Model adapted from [2] and [50].

An unexpected observation of our study was the behavior of the Her2/*neu*-targeted NiV-LV. In contrast to NiV-LVs targeted to CD8, EpCAM, or CD20, it was substantially reduced in mediating gene transfer lagging behind its MV-based counterpart by about 30-fold. Changes in cell surface expression, particle incorporation as well as Her2/*neu* binding could be excluded as being causative. Also blocking proteolytic degradation after potential endocytosis did not restore gene transfer activity to similar levels as that of the MV-based vector particles. Among a panel of six further Her2/*neu* binding domains, however, two DARPins and a scFv were identified that mediated substantially higher titers now being in the same range or even exceeding those of the corresponding MV-based LVs. Vector particles displaying these Her2/*neu* binding domains being active in mediating transduction neither contained more G protein nor were they more active in binding Her2/*neu*. Strikingly, their binding sites invariably localize to the membrane proximal domain IV of Her2/*neu*, while those of the four binding domains mediating low transduction rates localize to the membrane distal domain I (Fig 10A). Her2/*neu* is known to exist mainly in the so called “open” conformation in which the extracellular domains are straightened up and thus oriented almost perpendicularly to the cell membrane [48]. Thus, vector particles binding to the membrane distal domain I of Her2/*neu* will be about 80 Å further away from the cell membrane than those displaying a domain IV-specific targeting domain. The SCF binding site on CD117 (about 120 Å) and the ATD of GluA4 are similarly far away from the cell membrane [38,39]. Indeed, titers of the NiV-LV particles targeted to these receptors were in a similar low range as that of NiVmut^Her2^-LV, but increased by at least 20-fold when we moved domains I-III of CD117 and the ATD of GluA4 closer to the membrane (Fig 10A).

In contrast to CD117, GluA4 and Her2/*neu*, the natural NiV receptor ephrin-B2 is a transmembrane protein with a single, rather small extracellular domain that brings the bound virus particle similarly close to the cell membrane as domain IV of Her2/*neu*. This holds true also for EpCAM and CD20, which both mediated efficient entry of the targeted NiV-LV particles (Fig 10A). The extracellular part of CD8α is composed of a single immunoglobulin (Ig)-like domain linked to a thin stalk sequence of 47 residues. Although the 3D structure of the stalk is not available, it is assumed to be highly flexible, thus also allowing a close proximity of vector particles having attached to the Ig-like domain [49].

Taken together, we thus have experimental evidence from three different receptors that changing the distance of the attachment site relative to the plasma membrane makes a huge difference in particle entry. Thereby, it was irrelevant if we altered the distance by receptor engineering (CD117, GluA4) or by displaying targeting domains that bind to more membrane-proximal epitopes (Her2/*neu*). We can therefore conclude, with only some uncertainty for CD8, that gene delivery mediated by NiV-LVs at high efficiency requires binding of cell surface receptors close to the cell membrane within a maximal distance of about 50 Å. Receptor attachment in distances clearly beyond this results in a substantially reduced gene delivery efficiency, most likely due to inefficient or absent membrane fusion.

How can we imagine that the distance between attachment site and cellular membrane makes such a huge difference for pH-independent membrane fusion mediated by the NiV glycoproteins? It is important to realize that NiV-LV particles are completely covered with glycoproteins (Fig 2F). Thus upon cell attachment, a rigid scaffold will be formed between viral and cellular membranes by numerous glycoprotein-receptor contacts. These trigger conformational changes in G and F, which then projects the fusion peptide on top of a long coiled-coil structure, the heptad repeat A (HRA), towards the cell membrane [2]. In its fully extended, so called prehairpin intermediate state, F can cover a maximal distance of 210 Å between the viral and the cellular membrane [50]. Although the structure of the prehairpin intermediate has so far only been modeled for parainfluenza virus 5 (PIV5), we can assume a very similar distance for the NiV F protein, since structure and size of HRA and HRB (adjacent to the transmembrane domain) are well conserved among paramyxoviruses [51,52] and the recently crystallized prefusion form of NiV F exhibits an overall similar size as that of PIV5 [53]. With G protein being slightly bigger in size than F, and receptor attachment sites being located on top of the globular heads of G [25,26], we estimate the natural receptor binding site being about 120 Å away from the viral membrane. The conformational change in F can then cover an additional distance of up to 90 Å. Any distance beyond that would not allow insertion of the fusion peptide into the cell membrane. The distances we determined here for the G-receptor pairs which were inefficient in mediating vector particle entry were indeed above 90 Å. The most likely explanation for our observations thus is an incompatible architecture of the fusion protein with rigid receptors that expose binding sites for NiV more than 90 Å away from the cell membrane (Fig 10B).

While most of the paramyxoviruses use sialic acid as receptor and can thus choose between many attachment sites exposed at various distances from the cell membrane, Henipa- and morbilliviruses using protein receptors must have adapted to receptors bringing them so close to the cell membrane that the distance between both viral and cellular membrane can be covered by their F protein.

Supporting this model, a study analyzing a panel of chimeric CD46-CD4 proteins to function as MV receptors demonstrated that putting the MV binding domains of CD46 on top of the complete CD4 molecule (four extracellular Ig domains) strongly reduced membrane fusion [54]. While this fits nicely to our observations for the NiV glycoproteins, targeting MV-pseudotyped LVs to the membrane distal domain of Her2/*neu* did not affect gene delivery (Fig 5C) [30]. A prominent difference between MV-LVs on one hand and NiV-LVs as well as MV on the other is the level of incorporated glycoproteins. NiV-LVs are completely covered with glycoproteins (Fig 2F) as it is the case for MV particles [55]. MV-LV particles, in contrast, contained on average more than three-fold less H than NiV-LVs G protein. Thus, LV particles pseudotyped with MV glycoproteins bind to cells via very few or even single receptor contacts, which leaves them more flexibility to take a position within an optimal distance to the cell surface for membrane fusion. This may well help MV-LVs to better compensate when being bound to a membrane distal domain of a receptor. For NiV-LVs, in contrast, this may not be as easily possible since they form many receptor contacts resulting in a much more rigid complex between virus particle and target cell. Moreover, the henipavirus G proteins are unique among all paramyxoviruses, including MV, in forming covalently linked tetramers (dimers-of-dimers) [56]. This could further contribute to a more rigid receptor-attachment protein complex for NiV than for MV, which in turn results in higher sensitivity towards membrane-distal receptor attachment.

In summary, the data presented in this manuscript imply that for the engineering of cell-type-specific LVs, binding domains should be used bringing the particles within a close distance to the cell membrane. By applying this to NiV-LVs, important progress in the engineering of cell-type specific LVs has been made. Titers of these vectors are substantially enhanced compared to vectors pseudotyped with engineered MV glycoproteins. The reasons for this could be allocated to an increased number of particles released from packaging cells which is most likely due to the intrinsic budding capability of the NiV glycoproteins [57]. Second, the particles are more active in delivering the packaged gene which is likely the consequence of the substantially higher glycoprotein density of NiV-LV particles compared to MV-LVs. Since NiV-LVs can be produced at titers exceeding 10^6^ t.u./ml, they will better qualify for scale up and GMP production—an important requirement for applying receptor-targeted LVs in clinical settings.

## Materials and Methods

### Generation of constructs

The plasmid pHL3-Ac1 coding for truncated and mutated MV Hc∆18mut protein and for a (G_4_S)_3_ linker (L3) between H and the His-tagged DARPin Ac1 was generated by inserting the PCR-amplified coding sequence of the EpCAM specific DARPin Ac1 [21] from pQE30ss_Ac1_corr into the backbone of plasmid pHL3-HRS3opt2#2 [58] via *Sfi*I/*Not*I. All plasmids encoding Nipah virus G protein variants were derived from pCAGGS-NiV-G [59]. The coding sequence for the Ac1 targeting domain was fused to the C-terminus of the G protein reading frame by PCR amplification of each fragment and simultaneously introducing a common *AgeI* restriction site, which was used for ligation resulting in plasmid pCAGGS-NiV-G^EpCAM^. All other targeting domains (DARPins or scFv) were exchanged via *AgeI*/*Not*I, resulting in the corresponding expression plasmids encoding Gc∆34 fused to the targeting domain. Truncations of the G protein cytoplasmic tail were introduced by PCR of the G protein reading frame and insertion of the PCR fragments into pCAGGS-NiV-G^EpCAM^ resulting in plasmids pCAGGS-NiV-Gc∆33^EpCAM^ and pCAGGS-NiV-Gc∆34^EpCAM^.

The His-tagged G^His^ and Gc∆34^His^ proteins were generated by PCR amplification from pCAGGS-NiV-G. The fragments were cloned via *Pac*I/*Not*I restriction into the plasmid backbone of pCAGGS-NiV-G^EpCAM^ resulting in pCAGGS-NiV-G^His^ and pCAGGS-NiV-Gc∆34^His^, respectively. Mutations interfering with natural receptor recognition were introduced into the NiV-Gc∆34^EpCAM^ protein coding sequence by site-directed mutagenesis. Each mutation was generated by amplification of two fragments carrying the designated mutation with homologous regions at the mutation site. These fragments were fused and amplified by a flanking primer pair. Resulting fragments were cloned into pCAGGS-NiV-Gc∆34^EpCAM^ via *Rsr*II/*Age*I, generating the plasmids pCAGGS-NiV-Gc∆34^EpCAM^mut.

For the generation of the NiV-F variants, the coding sequences for Fc∆22 [60] and Fc∆25 were amplified from pCAGGS-NiV-F [59] and cloned via *Pac*I/*Sac*I restriction into the plasmid backbone of pCAGGS-NiV-G resulting in the plasmids pCAGGS-NiV-Fc∆22 and pCAGGS-NiV-Fc∆25. AU1 tagged NiV-F variants used for Western blot analysis of vector particles were generated by amplifying the NiV-F variants from pCAGGS-NiV-F and simultaneously adding the AU1 tag C-terminally. The resulting PCR fragments were cloned via *Pac*I/*Sac*I into the backbone of pCAGGS-NiV-G, resulting in the plasmids pCAGGS-NiV-F-AU1, pCAGGS-NiV-Fc∆22-AU1, and pCAGGS-NiV-Fc∆25-AU1.

For sequences of primers used for the PCR reactions see S1 Table.

### Cell culture

HEK-293T (ATCC CRL-11268), U87-MG (ATCC HTB-14) and CHO-K1 (ATCC CCL-61) cells were grown in Dulbecco’s modified Eagle’s medium (DMEM) (Sigma-Aldrich, Munich, Germany) supplemented with 10% fetal calf serum (FCS) (Biochrom, Berlin, Germany) and 2 mM _L_-glutamine (Sigma-Aldrich, Munich, Germany). SK-OV-3 (ATCC HTB-77) cells were grown in McCoy’s A5 medium (Sigma-Aldrich, Munich, Germany) supplemented with 10% FCS and 1% _L_-glutamine. Raji (ATCC CCL-86) as well as Molt4.8 cells were grown in RPMI 1640 (Biowest, Nuaillé, France) supplemented with 10% FCS and 2 mM _L_-glutamine.

The cell lines CHO-EpCAM [61] and CHO-ephrin-B2 were derived from CHO-K1 cells (ATCC CCL-61) and cultivated in the same medium in presence of 10 μg/ml puromycin (Thermo Fisher Scientific, Waltham, USA). For generation of CHO-ephrin-B2 cells, the gene encoding human ephrin-B2 was amplified from pCAGGS-EB2 [62] and cloned into a lentiviral transfer vector resulting in the bicistronic plasmid pS-ephrin-B2-IRES-puro-W. CHO-K1 cells were transduced with LV particles having packaged the ephrinB2-IRES-puro sequence and were selected using 10 μg/ml puromycin for 2 weeks.

HT1080-CD117 and HT1080-GluA4 cells were derived from HT1080 cells (ATCC CCL-121). For this, the coding sequence for human CD117 was amplified via PCR from pCMV6-XL4-cKIT (OriGene Technologies, Rockville, USA) and that of GluA4 from pk0002-Imyc [63] (kindly provided by Kari Keinänen), thereby adding an N-terminal myc tag. PCR fragments were then cloned into the backbone of pS-ephrin-B2-IRES-puro-W (*Bam*HI/*Spe*I) resulting in the bicistronic plasmids pS-CD117-IRES-puro-W and pS-GluA4-IRES-puro-W, respectively. For the shortened receptor versions, the coding sequence for domains I-III of human CD117, and of the amino terminal domain (ATD) of murine GluA4 were fused to that of the CD4 transmembrane domain, respectively, and cloned into the backbone of pS-ephrin-B2-IRES-puro-W (*Bam*HI/*Spe*I) resulting in pS-CD117short-IRES-puro-W and pS-GluA4short-IRES-puro-W. HT1080 cells were transduced with LVs having packaged the receptor encoding constructs and selected using 10 µg/ml puromycin for 2 weeks.

Primary PBMC were isolated from human buffy coats purchased from the German blood donation center (DRK-Blutspendedienst Hessen, Frankfurt). PBMC were activated for 72 h in RPMI 1640 supplemented with 10% FCS, 2 mM _L_-glutamine, 0.5% streptomycin/penicillin, 25 mM HEPES (Sigma-Aldrich, Munich, Germany), 100 U/ml interleukin-2 (R&D Systems, Minneapolis, USA), 1 μg/ml CD3 antibody (clone: OKT3, eBioscience, San Diego, USA) and 1 μg/ml CD28 antibody (clone: CD28.2, eBioscience, San Diego, USA). Following transduction, cells were cultivated in the same medium without OKT3 antibody and CD28 antibody.

### LV production and transduction

Vector particles were generated by transient transfection of HEK-293T cells using polyethylenimine (PEI). Twenty-four hours before transfection, 2.5x10^7^ cells were seeded into a T175 flask. On the day of transfection, the cell culture medium was replaced by 10 ml DMEM with 15% FCS and 3 mM _L_-glutamine. The DNA mix was prepared by mixing 35 μg of total DNA with 2.3 ml of DMEM without additives. For initial experiments (Fig 1B and 1D) 1.35 μg of plasmid DNA encoding NiV-G wildtype or truncation mutants was mixed with 4.04 μg plasmid DNA encoding the NiV-F variants, 14.5 μg of the packaging plasmid pCMV∆R8.9 [64] and 15.2 μg transfer vector pSEW encoding green fluorescent protein (GFP) as reporter [65]. Following optimization of G to F ratios, 0.9 μg of plasmid encoding G protein variants were mixed with 4.49 μg plasmid coding for F variants. The amounts of packaging plasmid and transfer vector remained unchanged. LVs pseudotyped with the VSV glycoprotein G were generated by co-transfecting cells with 6.13 μg pMD2.G (kindly provided by Didier Trono, Lausanne, Switzerland), 11.38 μg pCMV∆R8.9 and 17.5 μg pSEW. The transfection reagent mix was prepared by adding 140 μl of 18 mM PEI solution in H_2_O to 2.2 ml DMEM without additives. This solution was combined with the DNA mix, vortexed, incubated for 20 minutes at room temperature and added to the HEK-293T cells, resulting in DMEM with 10% FCS, 2 mM _L_-glutamine in total. 24 h later, the medium was replaced by DMEM with 10% FCS, 2 mM _L_-glutamine. At day two post transfection, cell supernatants containing the vector particles were passed through a 0.45 μm pore size filter. If needed, vector particles were purified by centrifugation at 4500 rpm for 24 h over a 20% sucrose cushion. The pellet was resuspended in phosphate-buffered saline (PBS).

For transduction, 8x10^3^ of CHO-EpCAM and SK-OV-3 cells or 2x10^4^ Molt4.8 and Raji cells were seeded into 96-well-plate and transduced on the next day. When needed, cells were pre-incubated for 30 min at 37°C with medium containing different concentrations of bafilomycin A1 (Santa Cruz Biotechnology, Dallas, USA) before LVs were added. For titration, cells were transduced with at least four serial dilutions of vector particles. After 72 h, the percentage of GFP-positive cells was determined by flow cytometry and the transducing units per milliliter (t.u./ml) were calculated by selecting the dilutions showing linear correlation between dilution factor and number of GFP-positive cells. For transduction of primary PBMC, cells and vector were spinfected by centrifugation at 850xg at 32°C for 90 minutes. Percentages of GFP-positive cells were determined by flow cytometry at the indicated days post transduction.

### Electron microscopy

For electron microscopy, concentrated NiVmut^EpCAM^-LV particles were adsorbed to glow discharged formovar coated 200-mesh nickel grids for 5 min, washed three times with H_2_O and contrasted with 2% aqueous uranyl acetate (Merck, Darmstadt, Germany) for 10 s. Samples were analyzed with the EM109 transmission electron microscope (Zeiss, Jena, Germany).

### Flow cytometry

Flow cytometry analysis was performed on the MACSQuant Analyzer 10 (Miltenyi Biotec, Bergisch Gladbach, Germany). For surface expression experiments of Nipah virus G constructs, HEK-293T cells were transfected with the corresponding expression plasmid. After 48 h, adherent cells were detached with PBS-EDTA solution and subsequently washed in 800 µl FACS washing buffer (PBS, 2% FCS, 0.1% NaN_3_), and incubated with a phycoerythrin (PE)-conjugated mouse anti-His antibody (clone GG11-8F3.5.1, Miltenyi Biotec, Bergisch Gladbach, Germany, dilution 1:100) in FACS washing buffer. Human EpCAM was detected by an Allophycocyanin (APC) labeled mouse anti-EpCAM antibody (clone HEA-125, Miltenyi Biotec, Bergisch Gladbach, Germany, dilution 1:100). CD117 and CD117short expression was detected by staining with PE-coupled CD117 antibody (clone: 104D2; 1:100; BioLegend, San Diego, USA). Expression of myc-tagged GluA4 and GluA4short was detected by staining with PE-coupled anti-myc antibody (clone 9B11; 1:100; Cell Signaling Technology, Danvers, USA). Primary PBMC were transferred into FACS washing buffer, washed twice and CD8 expression was detected by a human APC-conjugated anti-CD8 antibody (clone RPA-T8, 1:100, BD Biosciences, San Jose, USA). After two additional washing steps, cells were resuspended in 100 µl PBS containing 1% formaldehyde. Data were analyzed using FCS Express version 4.0 (De Novo Software, Glendale, USA).

### Binding assay

1.4x10^6^ HEK-293T cells were seeded into one well of a 6-well plate and transfected on the next day with 1.92 µg plasmid DNA coding for the different NiV-G constructs. 48 h later, cells were detached and 1x10^5^ cells were washed with FACS washing buffer, incubated with 1 µg of the Fc-tagged extracellular domain of human ephrin-B2, ephrin-B3 or Her2/*neu* (R&D Systems, Minneapolis, USA) for 1 h at 4°C, washed again and subsequently stained with a FITC-tagged anti-human Fc antibody (1:100, SouthernBiotech, Birmingham, USA). Samples were analyzed by flow cytometry.

### Competition assay

NiVmut^EpCAM^-LV, MV^EpCAM^-LV, VSV-LV and NiVwt-LV were pre-incubated for 1 h at 4°C with different amounts of soluble extracellular domains of human or murine EpCAM (Sino Biological, Beijing, China) or human ephrin-B2 and B3 (R&D Systems., Minneapolis, USA), respectively. Then, CHO-EpCAM or CHO-ephrin-B2 cells were transduced with pre-incubated LVs before GFP expression was analyzed after 72 h by flow cytometry.

### Neutralization assay

CHO-EpCAM and CHO-ephrin-B2 cells were transduced at a multiplicity of infection (MOI) of 0.4 with NiVmut^EpCAM^-LV, MV^EpCAM^-LV, VSV-LV and NiVwt-LV that have been pre-incubated with serial dilutions of intravenous immunoglobulins (IVIG, Intratect, Biotest, Dreieich, Germany) for 2 h at 37°C. After 72 h, the percentage of GFP-positive cells was determined by flow cytometry.

### Nanoparticle tracking analysis of lentiviral particles

Particle size and concentration of LVs was determined using the NanoSight NS500 instrument (Malvern Instruments, Worcestershire, UK). Concentrated vector stocks were diluted in degassed PBS to contain between 1x10^7^ and 1x10^9^ particles/ml and measured five times for 90 s at 25°C. NTA2.3 software (Malvern Instruments, Worcestershire, UK) was used for particle identification, size analysis and determination of particle concentration.

### Western blot analysis

Concentrated vector particles were denatured by incubation with 2x urea sample buffer (5% sodium dodecyl sulfate, 8 mM urea, 200 mM Tris-HCl, 0.1 mM EDTA, 0.03% bromphenol blue, 2.5% dithiothreitol, pH 8.0) for 10 minutes at 95°C, separated by gel electrophoresis on 10% sodium dodecyl sulfate-polyacrylamid electrophoresis gels, and blotted onto nitrocellulose membranes (GE Healthcare, Freiburg, Germany). Blots were incubated with mouse anti-His (clone 27E8, 1:1,000; Cell Signaling Technology, Danvers, USA) for detection of His-tagged NiV and MV glycoproteins, mouse anti-p24 (clone 38/8.7.47, 1:1,000; Gentaur, Aachen, Germany) for detection of the LV core protein p24 or goat anti-AU1 antibody for detection of AU1-tagged NiV F protein (1:1,000; Thermo Fisher Scientific, Waltham, USA). Subsequently, horseradish peroxidase conjugated secondary antibodies (1:2,000; DakoCytomation, Hamburg, Germany) were used and signals were detected using the ECL Plus Western Blotting Detection System (Thermo Fisher Scientific, Waltham, USA). Released photon units were then quantified using the IVIS Spectrum (PerkinElmer, Waltham, USA). To determine the photon intensities of bands corresponding to the glycoproteins and to p24, areas corresponding to the respective bands were manually defined using the Living Image 4.3.1 software (PerkinElmer, Waltham, USA). Background activities were identified by the negative control samples and subtracted from the glycoprotein and p24 signals.

### Ethics statement

Buffy-oats obtained from anonymous blood donors were purchased from the German blood donation center.

## Supporting Information

S1 Fig Scatter dot blot of fluorescence intensities of Fig 1C. (PDF)

S2 Fig Quantification of western blot Fig 1D. (PDF)

S3 Fig Scatter dot blot of fluorescence intensities of Fig 5A. (PDF)

S4 Fig Quantification of western blot Fig 5B
(PDF)

S5 Fig Particle concentration of lentiviral vector stocks shown in Fig 5C. (PDF)

S6 Fig Binding of recombinant Her2/*neu*. (PDF)

S7 Fig Surface expression of Her2/*neu* DARPin displaying G proteins. (PDF)

S8 Fig Quantification of LV particle incorporation levels of Her2-targeted G protein variants. (PDF)

S9 Fig Binding of recombinant Her2/*neu* to Gc∆34^Her2^mut4 variants. (PDF)

S10 Fig CD117- and GluA4-targeted NiV-LV. (PDF)

S1 Table PCR primers for cloning. (PDF)
