# Supplementary material for: Receptor-Targeted Nipah Virus Glycoproteins Improve Cell-Type Selective Gene Delivery and Reveal a Preference for Membrane-Proximal Cell Attachment
Source: PLoS Pathog. 2016 Jun 9;12(6):e1005641. doi: 10.1371/journal.ppat.1005641 (PMC4900575; doi:10.1371/journal.ppat.1005641)
Supplement: S1 Fig — (PDF) [file ppat.1005641.s001.pdf]

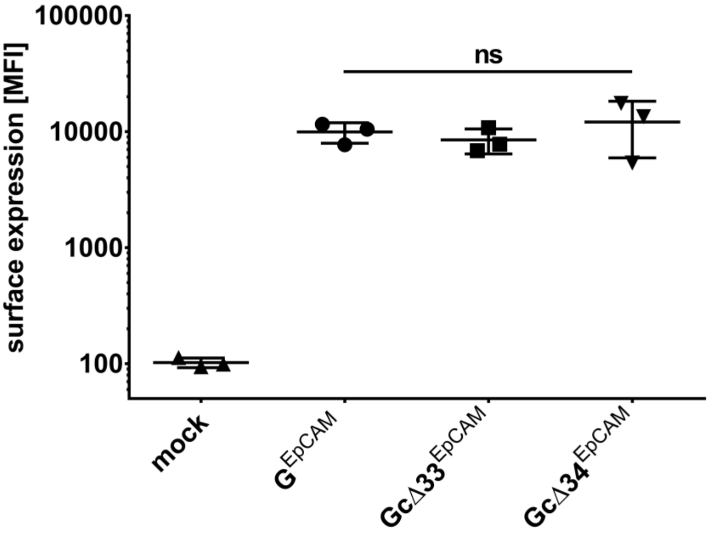

Figure S1: Scatter dot blot of fluorescence intensities of Fig 1C. Mean fluorescence intensities of HEK-293T cells transiently transfected with expression plasmids encoding the indicated EpCAM-displaying cytoplasmic tail truncation variants of G compared to mock transfected cells as determined by flow cytometry. Cells were stained with PE-coupled anti-His antibody (n=3; mean ± standard deviations (SD) are shown; ns, not significant by one-way ANOVA with Tukey's multiple comparisons test).
