# Supplementary material for: Receptor-Targeted Nipah Virus Glycoproteins Improve Cell-Type Selective Gene Delivery and Reveal a Preference for Membrane-Proximal Cell Attachment
Source: PLoS Pathog. 2016 Jun 9;12(6):e1005641. doi: 10.1371/journal.ppat.1005641 (PMC4900575; doi:10.1371/journal.ppat.1005641)
Supplement: S2 Fig — (PDF) [file ppat.1005641.s002.pdf]

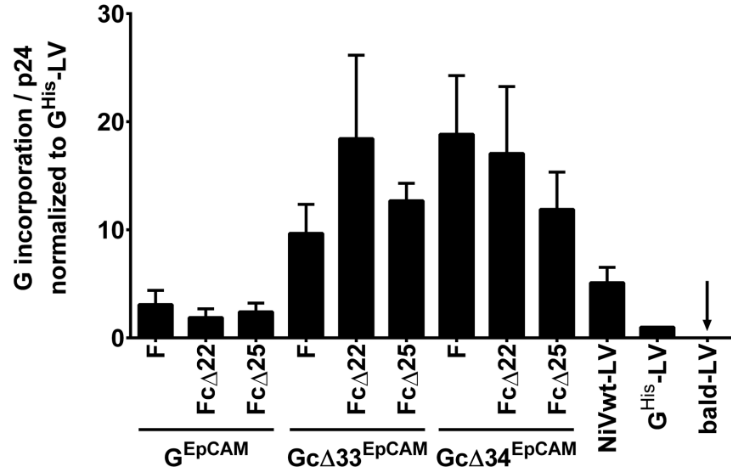

Figure S2: Quantification of Western blot Fig 1D. Western blot analysis of LV particles for incorporation of G^{EpCAM}, GcΔ33^{EpCAM}, and GcΔ34^{EpCAM} and the three different F variants (full length F, FcΔ22 and FcΔ25). $2.5x10^{10}$ particles per sample were applied. NiV-G^{His}/F (G^{His}-LV) and GcΔ34^{His}/FcΔ22 (NiVwt-LV) pseudotyped LVs as well as concentrated supernatant of mock transfected cells (mock) and concentrated supernatant of cells transfected with the *gag/pol* encoding plasmid pCMVΔR8.9 (bald-LV) served as control. Proteins were detected by chemiluminescense. Chemiluminescense values for the glycoprotein G variants were normalized to those of p24. Values are shown relative to that of G^{His}-LV. Data are mean values obtained from three independently generated vector stocks for each indicated envelope protein combination (n=3; mean ± standard error of the mean (SEM) are shown).
