# Supplementary material for: Receptor-Targeted Nipah Virus Glycoproteins Improve Cell-Type Selective Gene Delivery and Reveal a Preference for Membrane-Proximal Cell Attachment
Source: PLoS Pathog. 2016 Jun 9;12(6):e1005641. doi: 10.1371/journal.ppat.1005641 (PMC4900575; doi:10.1371/journal.ppat.1005641)
Supplement: S3 Fig — (PDF) [file ppat.1005641.s003.pdf]

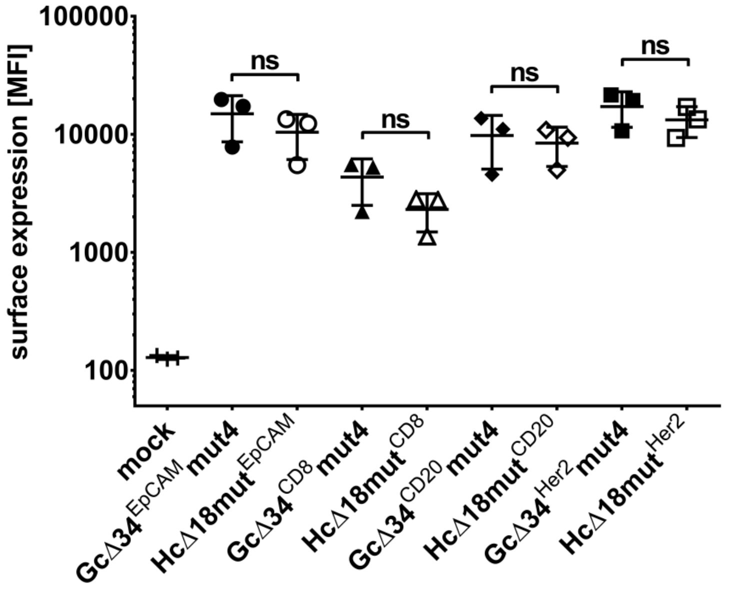

Figure S3: Scatter dot blot of fluorescence intensities of Fig 5A. Mean fluorescence intensities of surface expression of NiV G proteins targeted to four different receptors were compared to those of their corresponding MV H protein counterparts. All expression plasmids encoding the different constructs were transfected into HEK-293T cells. Surface expression was analyzed after 48 hours using a His-tag-specific antibody. Mock transfected cells served as negative control (n=3; mean ± standard deviations (SD) are shown; ns, not significant by unpaired *t*-test).
