# Supplementary material for: Receptor-Targeted Nipah Virus Glycoproteins Improve Cell-Type Selective Gene Delivery and Reveal a Preference for Membrane-Proximal Cell Attachment
Source: PLoS Pathog. 2016 Jun 9;12(6):e1005641. doi: 10.1371/journal.ppat.1005641 (PMC4900575; doi:10.1371/journal.ppat.1005641)
Supplement: S4 Fig — (PDF) [file ppat.1005641.s004.pdf]

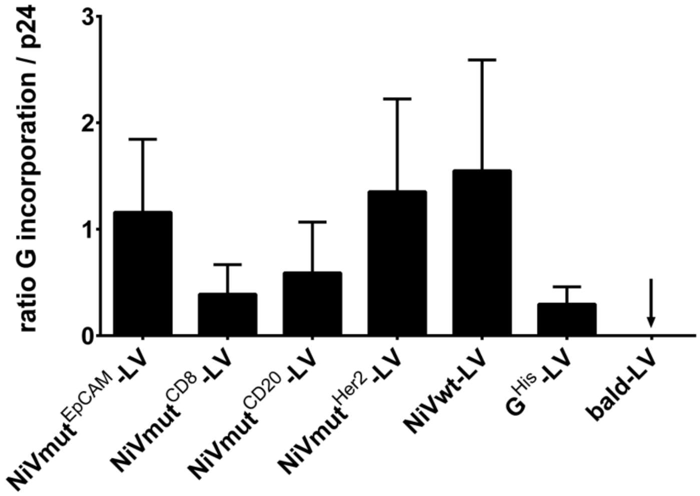

Figure S4: Quantification of Western blot Fig 5B. Three independently generated stocks of $NiVmut^{EpCAM}$-LV, $NiVmut^{CD8}$-LV, $NiVmut^{CD20}$-LV, and $NiVmut^{Her2}$-LV, were subjected to Western blot analysis applying $2.5x10^{10}$ particles per sample, respectively. Stocks prepared from mock transfected cells (mock) as well as stocks containing bald particles without glycoproteins (bald-LV) served as controls. In addition, particles pseudotyped with full-length His-tagged G and AU1 tagged F (G^{His}-LV) as well as particles pseudotyped with $Gc\Delta34^{His}/Fc\Delta22$-AU1 (NiVwt-LV) were used. Proteins were detected by chemiluminescense. Chemiluminescense values for the glycoprotein G variants were normalized to those of p24 (n=3; mean ± standard error of the mean (SEM) are shown).
