# Supplementary material for: Receptor-Targeted Nipah Virus Glycoproteins Improve Cell-Type Selective Gene Delivery and Reveal a Preference for Membrane-Proximal Cell Attachment
Source: PLoS Pathog. 2016 Jun 9;12(6):e1005641. doi: 10.1371/journal.ppat.1005641 (PMC4900575; doi:10.1371/journal.ppat.1005641)
Supplement: S5 Fig — (PDF) [file ppat.1005641.s005.pdf]

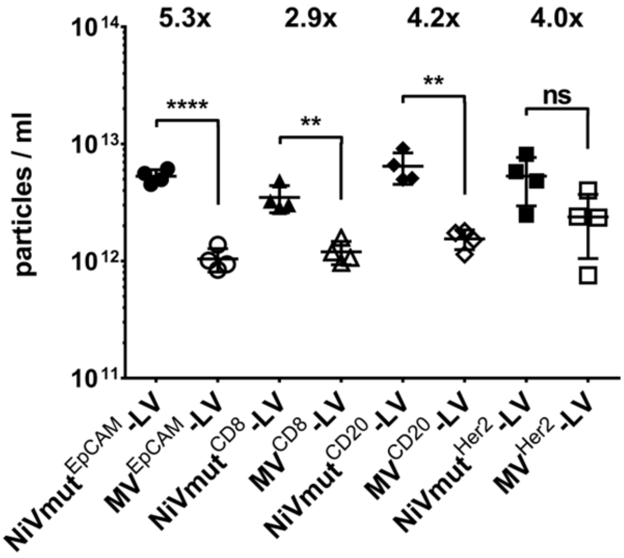

Figure S5: Particle concentration of lentiviral vector stocks shown in Fig 5C. The numbers of physical particles per ml were determined by single nanoparticle tracking analysis (NTA). The fold increase of particle numbers for the NiV-LV based stocks compared to the corresponding MV-LV based stocks are indicated. For each vector type mean values of four independently generated vector stocks are shown (n=4; mean ± standard deviations (SD) are shown; **, P<0.01; ****, P<0.0001; ns, not significant by unpaired *t*-test).
