# Supplementary material for: Receptor-Targeted Nipah Virus Glycoproteins Improve Cell-Type Selective Gene Delivery and Reveal a Preference for Membrane-Proximal Cell Attachment
Source: PLoS Pathog. 2016 Jun 9;12(6):e1005641. doi: 10.1371/journal.ppat.1005641 (PMC4900575; doi:10.1371/journal.ppat.1005641)
Supplement: S6 Fig — (PDF) [file ppat.1005641.s006.pdf]

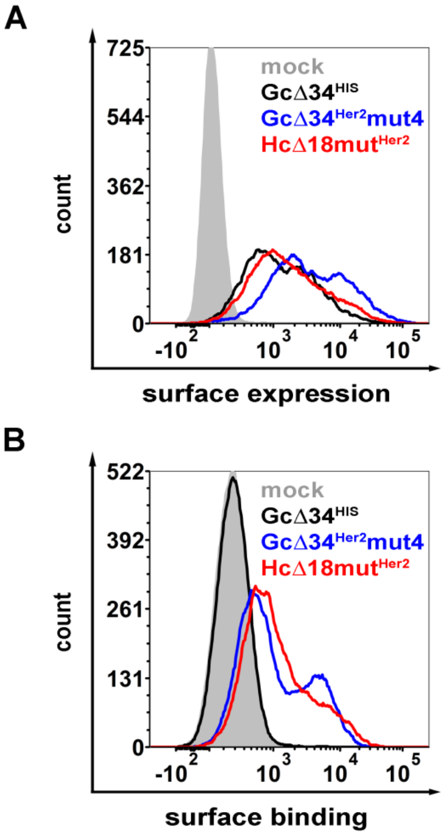

Figure S6: Binding of recombinant Her2/*neu*. (A) Exemplary flow cytometry blot of surface expression of NiV-GcΔ34^{Her2}mut4 (blue line) and HcΔ18mut^{Her2} (red line) in comparison to NiV-GcΔ34^{His} (black line). HEK-293T cells were transiently transfected with plasmids encoding the different glycoproteins compared to mock transfected cells (filled curve) as determined by flow cytometry. Cells were stained with PE coupled anti-His antibody. One representative out of three experiments is shown. (**B**) NiV-GcΔ34^{Her2}mut4 (blue line), HcΔ18mut^{Her2} (red line) and NiV-GcΔ34^{His} (black line) were expressed on HEK-293T cells, incubated for 1 h at 4°C with 1 µg/ml recombinant Fc-Her2/*neu* prior to staining against Fc-tag using FITC coupled anti-Fc antibody. Mock transfected cells (filled curve) served as control. One representative out of three experiments is shown.
