# Supplementary material for: Receptor-Targeted Nipah Virus Glycoproteins Improve Cell-Type Selective Gene Delivery and Reveal a Preference for Membrane-Proximal Cell Attachment
Source: PLoS Pathog. 2016 Jun 9;12(6):e1005641. doi: 10.1371/journal.ppat.1005641 (PMC4900575; doi:10.1371/journal.ppat.1005641)
Supplement: S7 Fig — (PDF) [file ppat.1005641.s007.pdf]

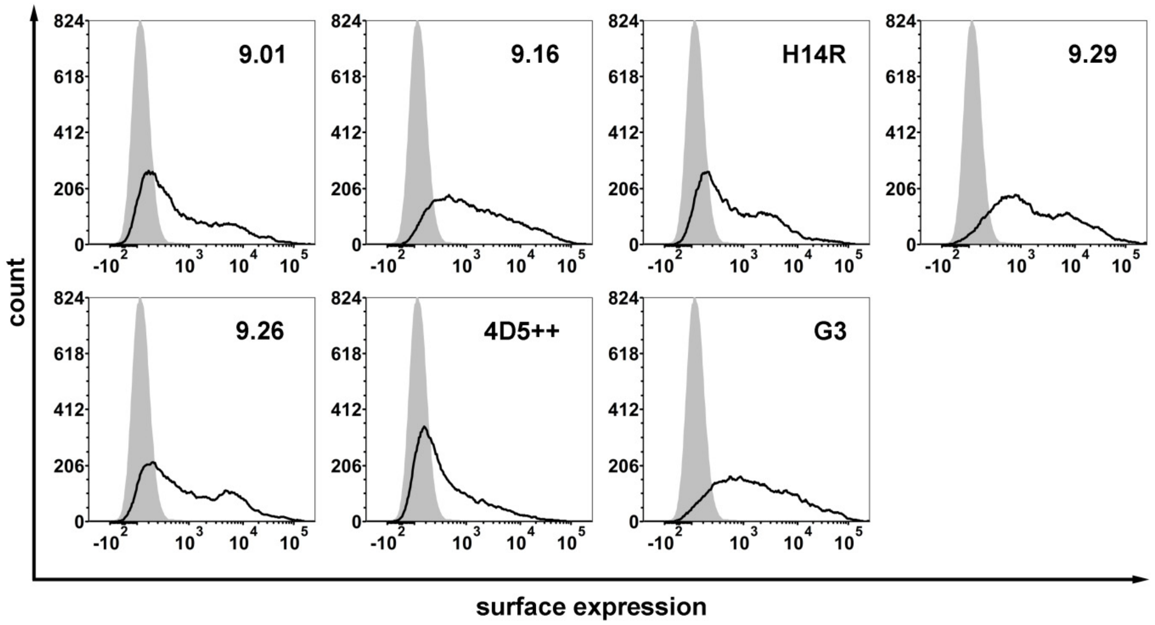

Figure S7: Surface expression of Her2/*neu* DARPin displaying G proteins. Representative flow cytometry plots showing the cell surface expression of GcΔ34^{Her2}mut4 variants fused to the indicated Her2/*neu*-specific DARPins (9.01, 9.16, H14R, 9.29, 9.26, G3) or the scFv 4D5++ after transient transfection of HEK-293T cells with the corresponding expression plasmids (empty curves) compared to mock transfected cells (filled curves). Cells were stained with PE-coupled anti-His antibody.
