# Supplementary material for: Receptor-Targeted Nipah Virus Glycoproteins Improve Cell-Type Selective Gene Delivery and Reveal a Preference for Membrane-Proximal Cell Attachment
Source: PLoS Pathog. 2016 Jun 9;12(6):e1005641. doi: 10.1371/journal.ppat.1005641 (PMC4900575; doi:10.1371/journal.ppat.1005641)
Supplement: S8 Fig — (PDF) [file ppat.1005641.s008.pdf]

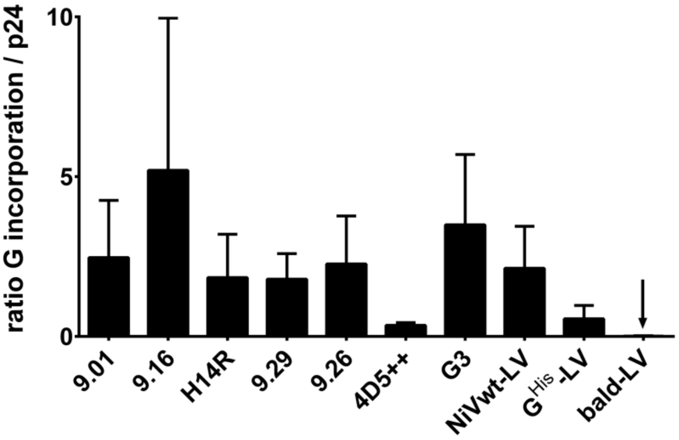

Figure S8: Quantification of LV particle incorporation levels of Her2-targeted G protein variants. The data refer to the exemplary Western blot shown in Fig 8G. Chemiluminescence values for the glycoprotein G variants and those of p24 were quantified from three Western blots and three independently generated vector stocks, respectively. Values for the G variants normalized to those of p24 are shown (n=3; mean ± standard error of the mean (SEM) are shown).
