# Supplementary material for: Receptor-Targeted Nipah Virus Glycoproteins Improve Cell-Type Selective Gene Delivery and Reveal a Preference for Membrane-Proximal Cell Attachment
Source: PLoS Pathog. 2016 Jun 9;12(6):e1005641. doi: 10.1371/journal.ppat.1005641 (PMC4900575; doi:10.1371/journal.ppat.1005641)
Supplement: S9 Fig — (PDF) [file ppat.1005641.s009.pdf]

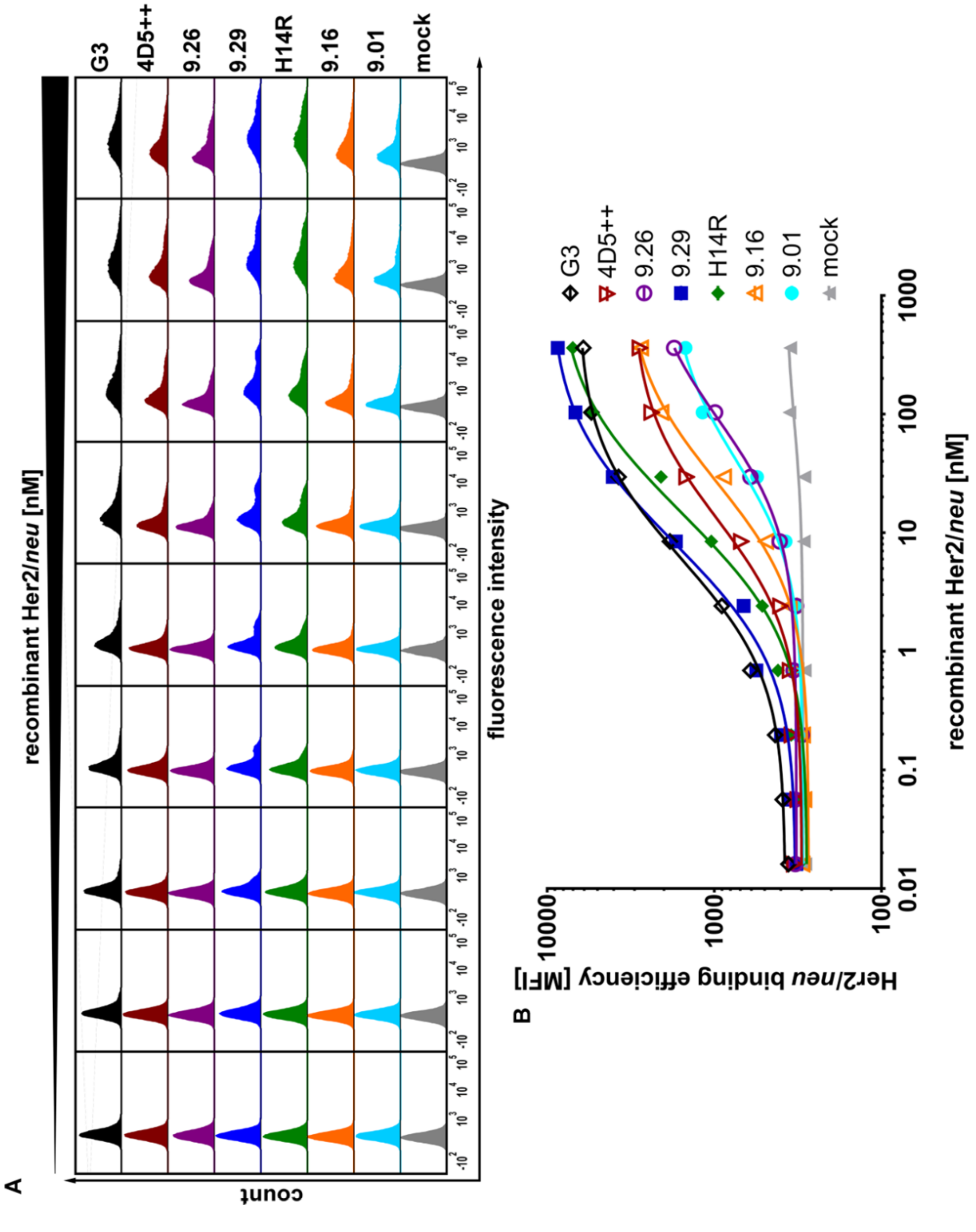

Figure S9: Binding of recombinant Her2/*neu* to GcΔ34^{Her2}mut4 variants. (**A**) Flow cytometry plots obtained after transient transfection of HEK-293T cells with expression plasmids encoding GcΔ34^{Her2}mut4 variants displaying either DARPin G3 (black), 9.26

(purple), 9.29 (blue), H14R (green), 9.16 (orange), 9.01 (cyan), or the scFv 4D5++ (red) and incubation for 1 h at 4°C with increasing molar concentrations of recombinant Fc-Her2/*neu* prior to staining against Fc-tag using FITC coupled anti-Fc antibody. Mock transfected cells were included as control (mock). (**B**) MFIs of the complete cell populations shown in (**A**) are plotted against the concentrations of recombinant Her2/*neu* applied for binding.
