# Supplementary material for: Receptor-Targeted Nipah Virus Glycoproteins Improve Cell-Type Selective Gene Delivery and Reveal a Preference for Membrane-Proximal Cell Attachment
Source: PLoS Pathog. 2016 Jun 9;12(6):e1005641. doi: 10.1371/journal.ppat.1005641 (PMC4900575; doi:10.1371/journal.ppat.1005641)
Supplement: S10 Fig — (PDF) [file ppat.1005641.s010.pdf]

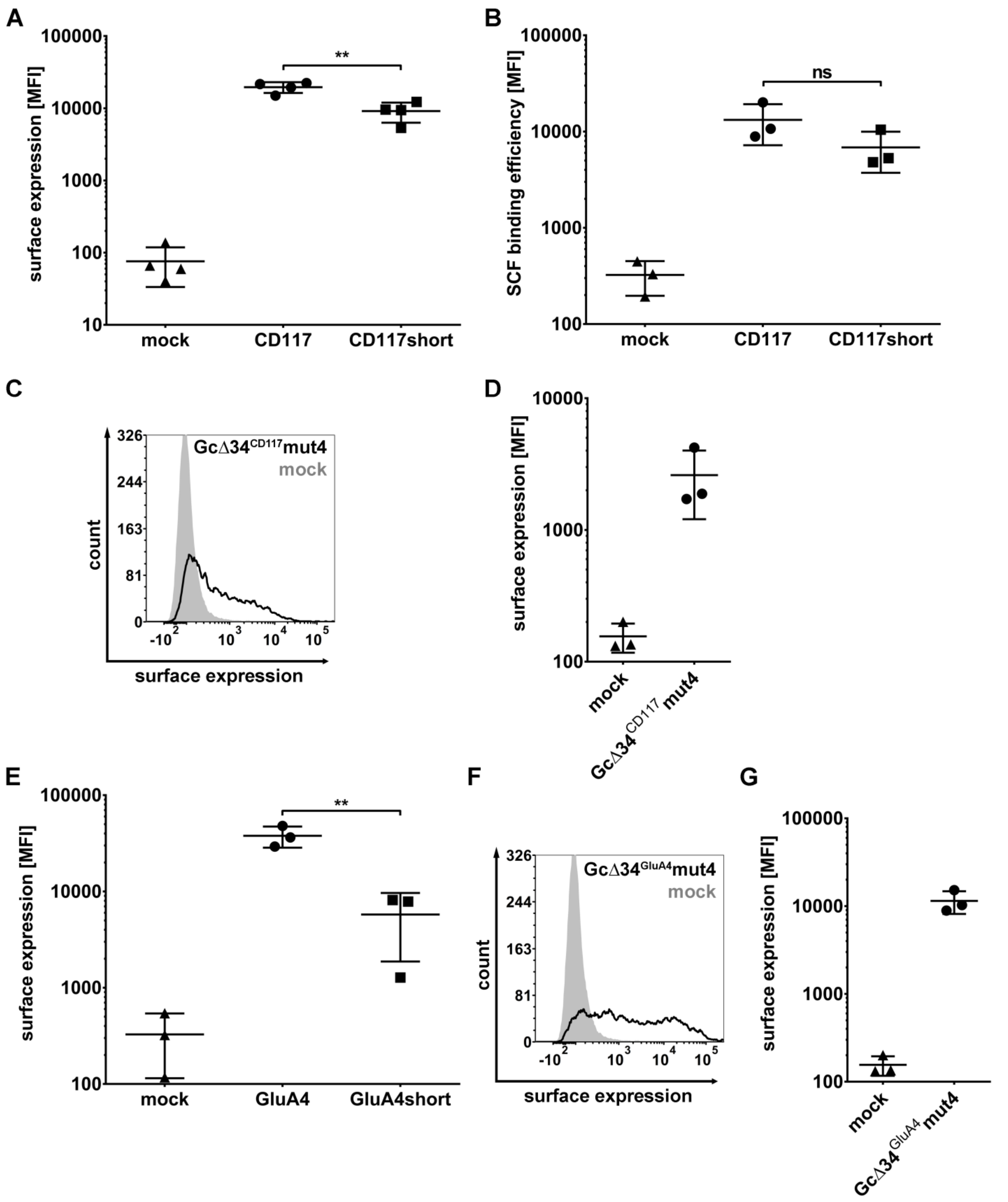

Figure S10: CD117- and GluA4-targeted NiV-LV. (**A**) Scatter dot blot of fluorescence intensities of Fig 9A. Mean fluorescence intensities of surface expression of CD117 and CD117short on HT1080 are shown. Surface expression was analyzed using a CD117-

specific antibody. Parental HT1080 cells served as negative control (mock) (n=4; mean ± standard deviations (SD) are shown; **, P<0.01 by unpaired *t*-test). (**B**) Scatter dot blot of fluorescence intensities of Fig 9C. Mean fluorescence intensities of surface binding of Fc-SCF to HT1080-CD117 and HT1080-CD117short are shown. Surface binding was analyzed using a FITC coupled anti-Fc antibody. Parental HT1080 cells served as negative control (mock) (n=3; mean ± standard deviations (SD) are shown; ns, not significant by unpaired *t*-test). (**C**) Exemplary flow cytometry blot of surface expression of NiV-GcΔ34^{CD117}mut4 (black line). HEK-293T cells were transiently transfected with plasmids encoding the glycoprotein and compared to mock transfected cells (filled curve) as determined by flow cytometry. Cells were stained with PE coupled anti-His antibody. One representative out of three experiments is shown. (**D**) Scatter dot blot of fluorescence intensities in (**C**). Mean fluorescence intensities of surface expression of the CD117-targeted G variant on HEK-293T cells transiently transfected with the corresponding expression plasmid compared to mock transfected cells as determined by flow cytometry. Cells were stained with PE-coupled anti-His antibody (n=3; mean ± standard deviations (SD) are shown). (**E**) Scatter dot blot of fluorescence intensities of Fig 9B. Mean fluorescence intensities of surface expression of GluA4 and GluA4short on HT1080 are shown. Surface expression was analyzed using a myc-tag specific antibody. Parental HT1080 cells served as negative control (mock) (n=3; mean ± standard deviations (SD) are shown; **, P<0.01 by unpaired *t*-test). (**F**) Exemplary flow cytometry blot of surface expression of NiV-GcΔ34^{GluA4}mut4 (black line). HEK-293T cells were transiently transfected with plasmids encoding the glycoprotein and compared to mock transfected cells (filled curve) as determined by flow cytometry. Cells were stained with PE coupled anti-His antibody. One representative out of three experiments is shown. (**G**)

Scatter dot blot of fluorescence intensities in (**F**). Mean fluorescence intensities of surface expression of GluA4-targeted G variant on HEK-293T cells transiently transfected with the corresponding expression plasmid compared to mock transfected cells as determined by flow cytometry. Cells were stained with PE-coupled anti-His antibody (n=3; mean ± standard deviations (SD) are shown).
