# Supplementary material for: Receptor-Targeted Nipah Virus Glycoproteins Improve Cell-Type Selective Gene Delivery and Reveal a Preference for Membrane-Proximal Cell Attachment
Source: PLoS Pathog. 2016 Jun 9;12(6):e1005641. doi: 10.1371/journal.ppat.1005641 (PMC4900575; doi:10.1371/journal.ppat.1005641)
Supplement: S1 Table — (PDF) [file ppat.1005641.s011.pdf]

**Table S11: PCR primers for cloning**

| Name | Sequence (5' – 3') |
|---|---|
| AgeI-DARPin fwd | ATA TAC CGG TGA CCT GGG TAA GAA ACT GCT GGA AG |
| AgeI-DARPin-G3 fwd | ATA TAC CGG TGA CCT GGG TAA GAA ACT ACT GGA AGC |
| AgeI-$(G_4S)_3$ fwd | ATA TAC CGG TGG AGG TGG CTC TGG TG |
| AgeI-NiV-G rev | ATA TAC CGG TGC ACT GCT CGG GGA TC |
| AgeI-NiV-G(I588) rev | GGT CAC CGG TGC ACT GCT CGG GGA TCT TCA CGG CGA ACA GCT TGG GCC TGG CCA CGT TGT CGC CGG TAT CG |
| AgeI-scFv-CD20 fwd | ATA TAC CGG TAT GGC TCA GGT TCA GCT GGTC |
| BamHI-cKIT fwd | ATA TGG ATC CAC CGG TCG CCA CCA TGA GAG GCG CTC G |
| BamHI-ephrin-B2 fwd | TAT AGG ATC CGC CAC CAT GGC TGT GAG AAG GGA CTC C |
| BamHI-GluA4 fwd | ATA TGG ATC CAC CGG TCG CCA CCA TGA GGA TTA TTT GCA GG |
| BsiWI-CD4-TM fwd | ATA TCG TAC GTC GGG ACA GGT CC |
| cKIT-D3-BsiWI rev | ACC TCG TAC GTA CTA CTT CCA AGG TTG TTG |
| cKIT-SpeI rev | GGT GTA CTA GTT CAG ACA TCG TCG TGC |
| GluA4-SpeI rev | CAG TAC TAG TTT ATG GTA GGT CCG ATG |
| GluA4-ATD-BsiWI rev | GCC GCG TAC GCA TAT CTT GAA TCA AGA C |
| HIS-DARPin rev | GTG ATG GTG ATG GTG ATG AGA ACC TCT TG |
| NiV-F fwd | CGC GTT AAT TAA GCC ACC ATG GTA GTT ATA C |
| NiV-FcΔ22-AU1 rev | GTA CGA GCT CTT AGA TGT ACC TGT ACG TGT CGG TGT TTC TCT TTT TCT CAA C |
| NiV-FcΔ25-AU1 rev | ATA TGA GCT CTT AGA TGT ACC TGT ACG TGT CCT TTT TCT CAA CAA TGA TAA AAC TGA TAA ATG |
| NiV-F-AU1 rev | GAT CGA GCT CTT AGA TGT ACC TGT ACG TGT CTG TCC CAA TGT AGT AGA GAT C |
| NiV-G(E501+W504) fwd | CAA CAC CTG CCC CGC GAT CTG CGC GGA GGG CGT GTA C |
| NiV-G(E501+W504) rev | GTA CAC GCC CTC CGC GCA GAT CGC GGG GCA GGT GTT G |
| NiV-G(E501A) fwd | CAA CAC CTG CCC CGC GAT CTG CTG GGA GG |
| NiV-G(E501A) rev | CCT CCC AGC AGA TCG CGG GGC AGG TGT TG |
| NiV-G(E533A) fwd | CAA CCA GAC CGC CGC GAA TCC CGT GTT CAC |

| | |
|---|---|
| NiV-G(E533A) rev | GTG AAC ACG GGA TTC GCG GCG GTC TGG TTG |
| NiV-G(Q530A+E533A) fwd | CTG GAC TCC AAC GCG ACC GCC GCG AAT CCC GTG TTC AC |
| NiV-G(Q530A+E533A) rev | GTG AAC ACG GGA TTC GCG GCG GTC GCG TTG GAG TCC AG |
| NiV-G(Q530A) fwd | CTG GAC TCC AAC GCG ACC GCC GAG AAT CCC |
| NiV-G(Q530A) rev | GGG ATT CTC GGC GGT CGC GTT GGA GTC CAG |
| NiV-G(W504A) fwd | CGA GAT CTG CGC GGA GGG CGT GTA C |
| NiV-G(W504A) rev | GTA CAC GCC CTC CGC GCA GAT CTC G |
| NiV-G(Y389A) fwd | CCA TCA CCA AGT GCC AGG CCA GCA AGC CCG AGA AC |
| NiV-G(Y389A) rev | GTT CTC GGG CTT GCT GGC CTG GCA CTT GGT GAT GG |
| NotI-DARPin rev | ATA TGC GGC CGC ATT AAG CTT TTG C |
| NotI-NiV-G rev | ATA TGC GGC CGC GGT GCA CTG CTC GGG GAT CTT CAC |
| NotI-scFv-CD20rev | ATA TGC GGC CGC CTT CAG CTC CAG CTT GGT CCC AGC AC |
| PacI-NiV-GcΔ33 | GCA CTT AAT TAA GCC ACC ATG ATC AAG AAG ATC AAC GAG GGC CTG CTG |
| PacI-NiV-GcΔ34 | GCA CTT AAT TAA GCC ACC ATG AAG AAG ATC AAC GAG GGC CTG CTG |
| SacI-NiV-FcΔ22 | GGC CGA GCT CTT AGG TGT TTC TCT TTT TCT CAA CAA TG |
| SacI-NiV-FcΔ25 | GGC CGA GCT CTT ACT TTT TCT CAA CAA TGA TAA AAC TGA TAA ATG |
| SacI-NiV-G rev | ATA TGA GCT CTC AGG TGC ACT GCT CGG GGA TC |
| SpeI-CD4-TM rev | GCC GAC TAG TTC AAA TGG GGC TAC ATG |
| SpeI-ephrin-B2 rev | ATA TAC TAG TTT AGA CCT TGT AGT AAA TGT TCG CCG GGC TC |
